# Glycolipidomics of Liver Flukes and Host Tissues during Fascioliasis: Insights from Mass Spectrometry Imaging

**DOI:** 10.1021/acsinfecdis.4c00551

**Published:** 2024-11-07

**Authors:** David Luh, Parviz Ghezellou, Sven Heiles, Svenja Gramberg, Simone Haeberlein, Bernhard Spengler

**Affiliations:** † Institute of Inorganic and Analytical Chemistry, Justus Liebig University Giessen, Giessen 35392, Germany; ‡ Leibniz-Institut für Analytische Wissenschaften-ISAS-e.V., Dortmund 44139, Germany; § Lipidomics, Faculty of Chemistry, University of Duisburg-Essen, Essen 45141, Germany; ∥ Institute of Parasitology, Biomedical Research Center Seltersberg (BFS), Justus Liebig University Giessen, Giessen 35392, Germany

**Keywords:** host−pathogen interactions, trematodes, *Fasciola hepatica*, glycosphingolipids, high-resolution MALDI mass spectrometry imaging, nanoscale liquid chromatography

## Abstract

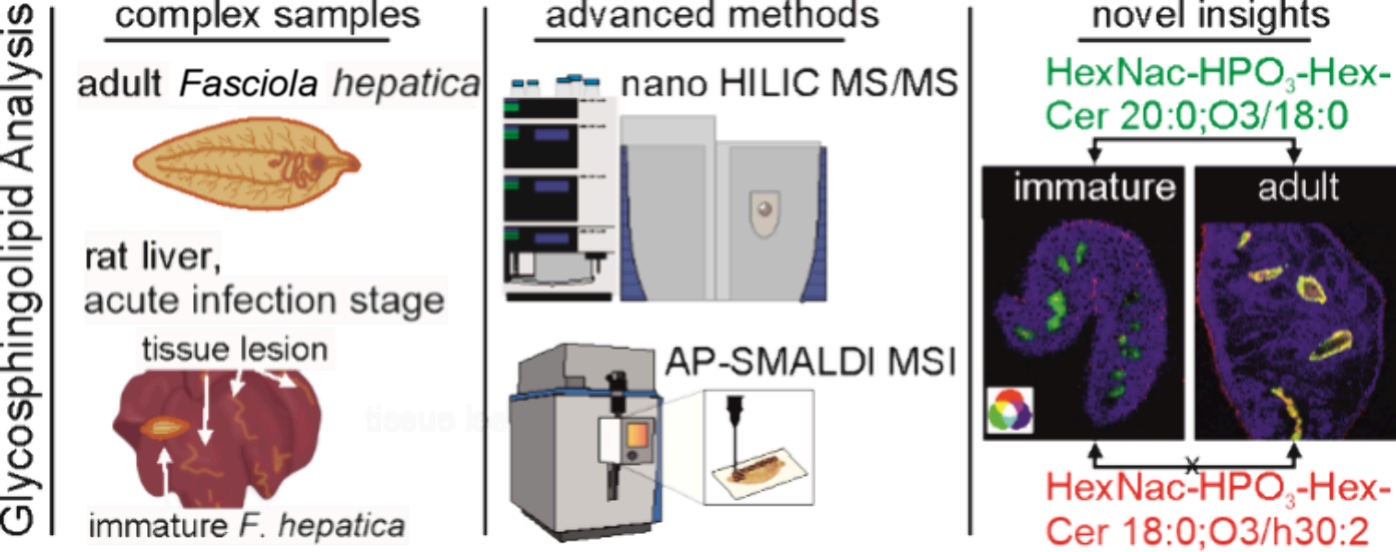

Fascioliasis, a zoonotic disease caused by liver flukes of the genus *Fasciola*, poses significant health threats to both humans and livestock. While some infections remain asymptomatic, others can lead to fatal outcomes, particularly during the acute phase characterized by the migration of immature parasites causing severe liver damage. Through the combination of data acquired via high-spatial-resolution atmospheric-pressure scanning microprobe matrix-assisted laser desorption/ionization mass spectrometry imaging (AP-SMALDI MSI) and nanohydrophilic interaction chromatography tandem mass spectrometry, we investigated glycosphingolipids (GSLs) in both adult and immature parasite stages as well as the host liver and bile duct to unravel the intricacies of the host–pathogen interplay and associated pathology. Several GSLs showed characteristic distribution patterns within the parasite depending on the fatty acid composition of their ceramides, notably including GSLs carrying very long-chain fatty acids. Additionally, GSL compositions within the tegument of immature versus adult parasites varied, suggestive of tissue remodeling upon maturation. AP-SMALDI MSI further enabled the identification of GSLs potentially involved in *in vivo* interactions between the host and immature parasites. Moreover, our experiments unveiled alterations in other lipid classes during *Fasciola* infection, providing a broader understanding of lipidomic changes associated with the disease. Collectively, our findings contribute to a deeper comprehension of the molecular intricacies underlying fascioliasis, with a specific focus on GSLs.

Fascioliasis is a foodborne trematode infection affecting livestock, resulting in huge economic losses.^1^ This zoonotic infection also poses a threat to human health, with an estimated 2.4 million people affected worldwide.^2^ Although classified as a neglected tropical disease (NTD) by the world health organization (WHO), fascioliasis is prevalent on all inhabited continents.

*Fasciola* (*F.*) spp. have a complex life cycle, which includes an intermediate snail host and a mammalian final host (sheep, cattle, and humans among others). The final host is infected by ingestion of encysted larvae (metacercariae), attached to vegetation or floating in water. Within the intestine, newly excysted juveniles hatch from the cysts and penetrate the intestinal wall to reach the liver. There, the immature worms migrate through and feed on the liver tissue until they have grown into adult (mature) flukes. Adults reside within the bile duct.^3^

The migration of immature parasites through the liver parenchyma during the acute stage of infection is causing most of the pathogenesis.^3^ Tissue damage can be caused mechanically or by digestive enzymes of the parasite and also by inflammatory reactions of the host.^3^ The severity of symptoms (fever, anemia, weight loss, abdominal pain, and death) as well as the extent of liver damage varies depending on the number of parasites, the host’s immune response, and other factors.^4^ In parallel, mechanisms of liver regeneration and repair are initiated, which may either restore liver function or lead to liver fibrosis and cirrhosis in severe cases.^5^ The chronic stage of infection, marked by adult worms present in the bile ducts, is often asymptomatic but can also be associated with signs of biliary obstruction and abdominal pain. Fascioliasis is mainly treated with triclabendazole, which is effective against adult and juvenile worms. However, emerging resistance against the drug necessitates the search for novel treatments.^6,7^ Understanding the fundamental biology of the parasite and the host immune response is therefore required.

The generation of comprehensive omics data sets for *Fasciola* spp. has accelerated the study of key molecules involved in the biology, pathogenicity, and virulence of liver flukes and has opened new avenues for the development of novel control strategies.^8^ For *Fasciola hepatica* (*F. hepatica*), it has been shown that there are clear differences in the excreted proteins during the developmental stages of the parasites.^9,10^ Furthermore, glycans from adult *F. hepatica* and recently from newly excysted juveniles were studied for their influence on the host immune response.^11,12^ For example, Rodríguez et al. demonstrated by comparison of lectin assays from *F. hepatica* glycans and their corresponding oxidized glycans (without function) that specific glycan components of *F. hepatica* are relevant for the release of interleukin 4 and 10.^11^ The huge variability of N- and O-glycans from newly excysted juveniles, recently analyzed by de Marco Verissimo et al. using liquid chromatography electrospray tandem mass spectrometry (LC ESI MS/MS), provides the fundamental to elucidate the role of glycans during the migratory phase of the parasite.^12^ However, limited data are available for glycosphingolipids (GSLs), a molecular class known to be generally involved in the host immune response.^13−15^ Studies from Wuhrer et al. utilizing several analytical methods, such as high-performance thin-layer chromatography, matrix-assisted laser desorption/ionization (MALDI) time-of-flight mass spectrometry (MS), ESI MS, immunostaining, and NMR analysis, suggest high antigenicity of GSLs from adult *F. hepatica* as well as a mimicry of mammalian glycosphingolipids.^16−18^ However, there is a lack of information about the localization of these glycosphingolipids, and no information about GSLs from the immature stage or from the host is available. In addition, *in situ* studies on parasites in the host tissue that examined the lipidome are rare.

Mass spectrometry is often utilized to study the molecular composition of biological samples. Coupled with separation methods like LC, even complex biological mixtures can be comprehensively analyzed. However, tissue regional information or cell-specific biomolecular alterations are not available from MS-based bulk analyses. For this purpose instead, mass spectrometry imaging (MSI) and most prominently MALDI MSI enable visualization of localized biomolecular events.^19,20^ Here, the local information on hundreds to thousands of intact biomolecules can be obtained simultaneously during a nontargeted analysis. Improvements over the past years now routinely facilitate MALDI MSI analyses with a <10 μm spatial resolution.^21^ A more advanced setup even enables a spatial resolution down to 1.4 μm, moving forward to routine single-cell analysis.^22^

By a combination of MALDI MSI and reversed-phase LC tandem MS/MS (RP-LC MS/MS), the lipidome can be elucidated in detail. In addition, the combination with state-of-the-art nanohydrophilic interaction chromatography (nano-HILIC) MS/MS recently allowed us to globally and locally profile the GSLs of livers from *Schistosoma mansoni*-infected hamsters. Thereby, we revealed several GSLs potentially involved in immune response.^23^

In this study, we combined MALDI MSI and chromatography data to analyze various aspects of fascioliasis at the lipidomic level in depth. In this way, we hope to contribute to the fundamental understanding of the infection. For this purpose, different sample types from our rat model of both the chronic and acute infection stage were analyzed. A particular focus was set on the analysis of GSLs. The diversity of GSL species identified highlights a possible role during infection. We show that it is necessary to elucidate the structure of the GSLs at the molecular level as well as their distributions in the organism in order to propose new hypotheses. Additionally, state-of-the-art atmospheric-pressure scanning microprobe matrix-assisted laser desorption/ionization (AP-SMALDI) MSI experiments enabled an investigation of *in vivo* host–parasite interactions on a lipidomic level during the acute infection stage. We provide novel data, which can help to elucidate a small part of the complex interplay between the parasite and the host.

## Results and Discussion

### Study Design

**Figure 1:**
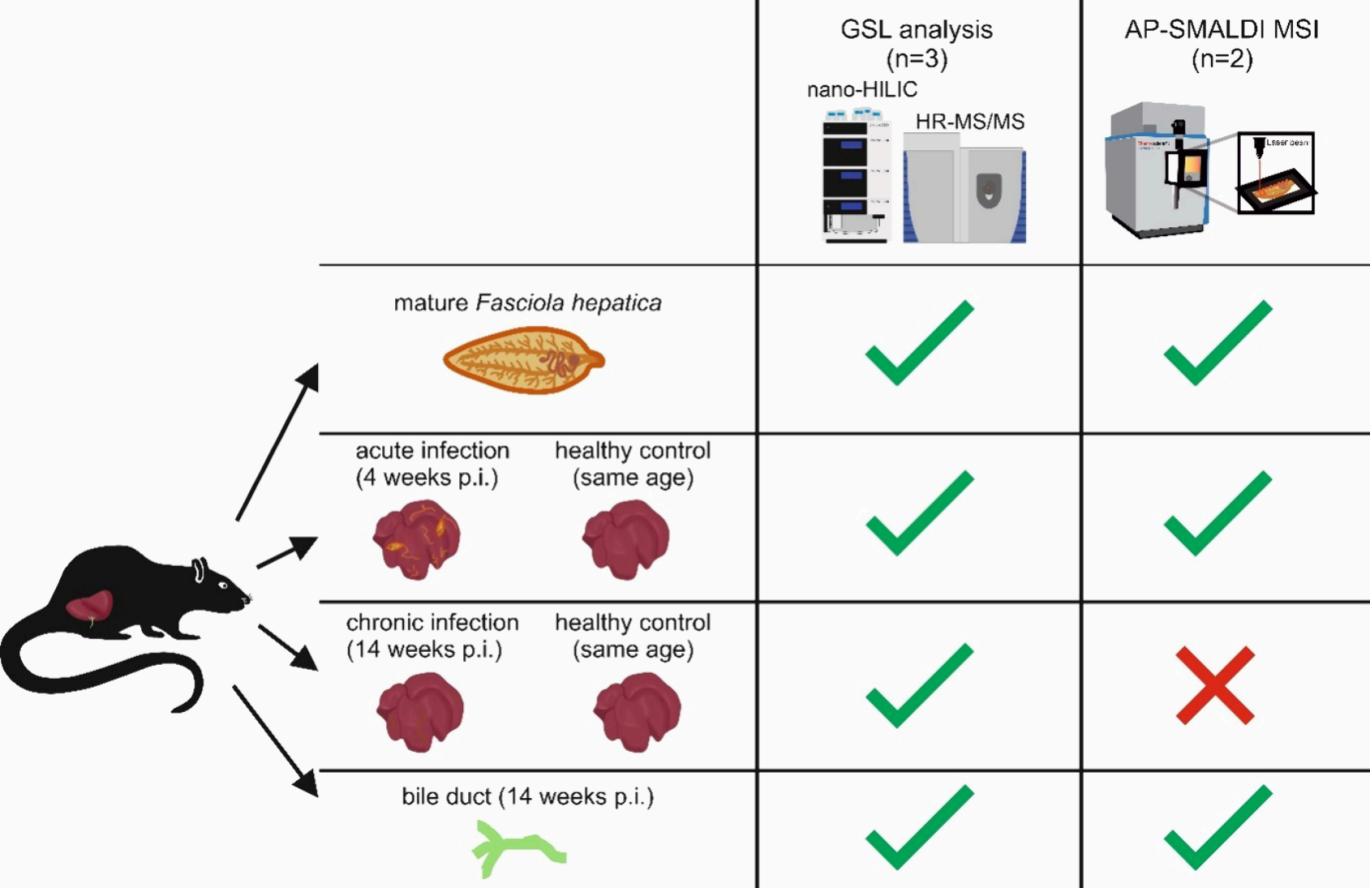
Overview of the study workflow. Adult *F. hepatica*were subjected to glycosphingolipid analysis, using nano-HLIIC MS/MS and AP-SMALDI MSI techniques. Rats were used as a model system for *F. hepatica* infection. Livers were collected 4 weeks post infection and 14 weeks post infection, representing the acute and chronic infection stage. For both stages, livers of uninfected rats of the same age served as control samples. In addition to noninfected controls, the bile duct of the chronic infection stage was also isolated and used for comparative glycosphingolipidome analysis using nano-HILIC MS/MS experiments. Furthermore, AP-SMALDI measurements were performed for rat livers at the acute infection stage and their corresponding control as well as bile duct samples. For chronic infection, tissue sections of rat livers were not subjected to AP-SMALDI analysis. “Tick” and “cross” indicate whether measurements were performed or not performed, respectively.

To comprehensively investigate the GSLs in isolated adult *F. hepatica* and parasites within the host organ, we employed nano-HILIC MS/MS and AP-SMALDI MSI. For the experiments, rat was used as an animal model. For global GSL characterization, nano-HILIC MS/MS was used, and the resulting curated databases guided AP-SMALDI MSI investigations. In order to study different stages of *F. hepatica* infection, livers after 4 weeks and after 14 weeks of infection were compared to control tissues (Figure 1). To minimize confounding variables of the control tissue, appropriate control samples with the same age of the rats, which were kept under the same conditions, were selected for the acute and chronic infection, respectively. These conditions were meant to probe the lipid composition during the acute (4 weeks) and chronic (14 weeks) infection phase in which *F. hepatica* are immature and adult, respectively. In addition, AP-SMALDI MSI measurements were performed with tissue sections of the rat liver during acute infection, which included migrating immature *F. hepatica* to examine *in vivo* host–parasite interactions. In the Supplementary Note 7 and Figure S1, we offer a detailed description of several different tissue areas in the liver of rats during the acute infection stage. As *F. hepatica* reside in bile ducts after maturation, no AP-SMALDI MSI of the liver from the chronic infection phase was performed, but MS imaging of isolated bile ducts was conducted. Three and two biological replicates were analyzed by nano-HILIC MS/MS and AP-SMALDI MSI measurements, respectively.

### Glycosphingolipids in Isolated Adult *F. hepatica*Parasites

**Figure 2:**
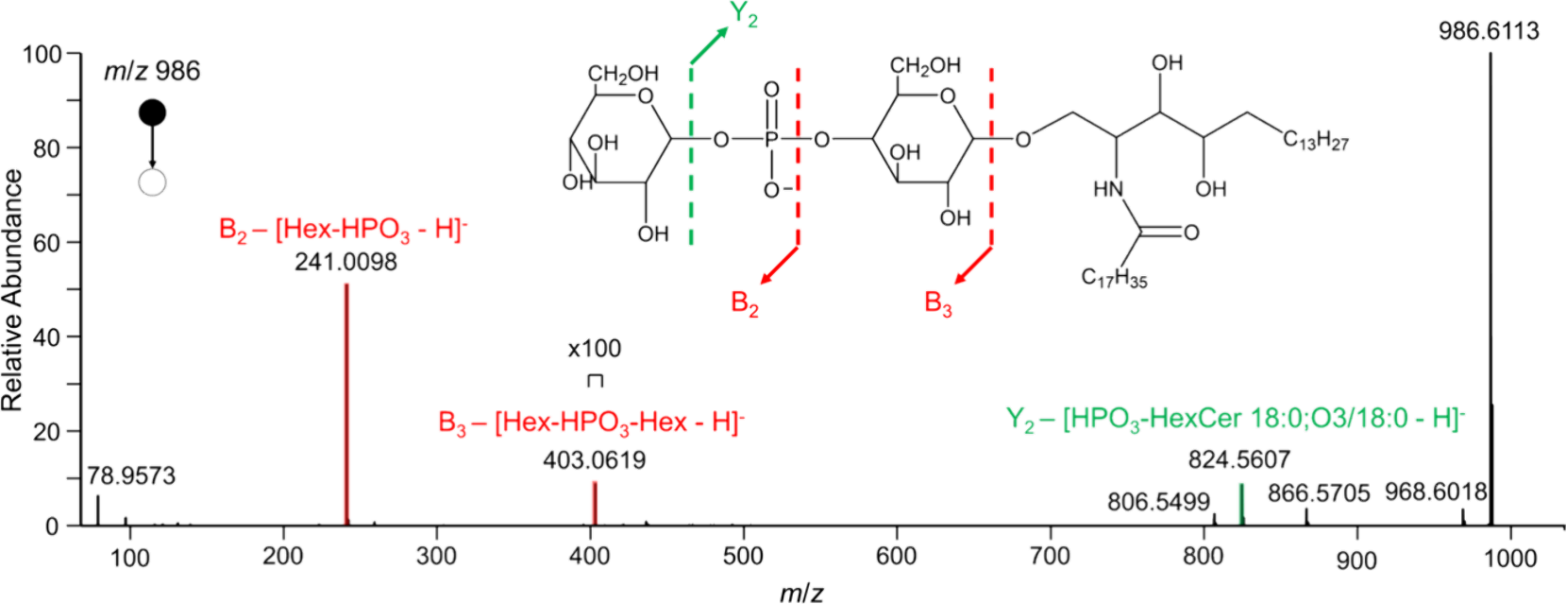
Tandem mass spectrum for Hex-HPO_3_-Hex-Cer 18:0;O3/18:0 ([M-H]^−^ at *m*/*z* 986.6113). The fragment ion at *m*/*z* 241.0098 represents the B_2_-fragment ion. The fragment ion at *m*/*z* 403.0619 corresponds to the B_3_-fragment ion. The fragment ion at *m*/*z* 824.5607 corresponds to the Y_2_-fragment ion.

To benchmark our nano-HILIC MS/MS method for parasites, we compared its performance on isolated adult *F. hepatica* with the GSL annotations reported for adult *F. hepatica* in previous studies, which were isolated without further *in vitro* incubation.^16−18^ We found that our GSL profile for neutral GSLs closely aligns with the species reported previously, with almost the same saccharide compositions and the same ceramide moieties identified (Table S11). This is an important indication that the short *in vitro* incubation of our isolated *F. hepatica* does not seem to profoundly affect the GSL profile. Apart from Hex_1_Cer, Hex_2_Cer, Hex_3_Cer, and Hex_5_Cer species, we also identified the presence of Hex_6_Cer species. Furthermore, our analysis was extended to intact GSLs, including the more intricate GSL-like Hex_3–4_HexNac_2–7_Cer, as listed in Table S11. For GSLs, variations in the ceramide backbone compositions were observed. We detected several phytosphingosines, but also, octadecasphinganine was consistently detected as part of the ceramide moiety in the GSLs throughout this study (Table S11). Notably, an *F. hepatica*-specific phosphate-containing GSL, a GlcNacα1-HPO_3_-6Gal(1–1) ceramide, with 14 different ceramide compositions, was identified as described by Wuhrer et al.^17^ In total, our analysis unveiled 35 GSL species containing a phosphate group, including various HPO_3_-Hex-Cer species with diverse ceramide backbones (a representative tandem mass spectrum is shown in Figure S2). In addition, we described for the first time the GSL Hex-HPO_3_-Hex-Cer for *F. hepatica*, with a representative tandem mass spectrum for this identified species being showcased in Figure 2. Fragment ions for this species are highlighted, such as the B_2_ and B_3_ ion with *m*/*z* 241.0098 and *m*/*z* 403.0619, respectively. Together with the Y_2_ ion (*m*/*z* 824.56070), we were able to determine the sequence. In comparison to the previously described GlcNacα1-HPO_3_-6Gal(1–1) ceramides, the newly discovered GSLs differ in the terminal saccharide unit.

### Distribution Patterns of Different Glycosphingolipids in Adult *F. hepatica*Parasites

**Figure 3:**
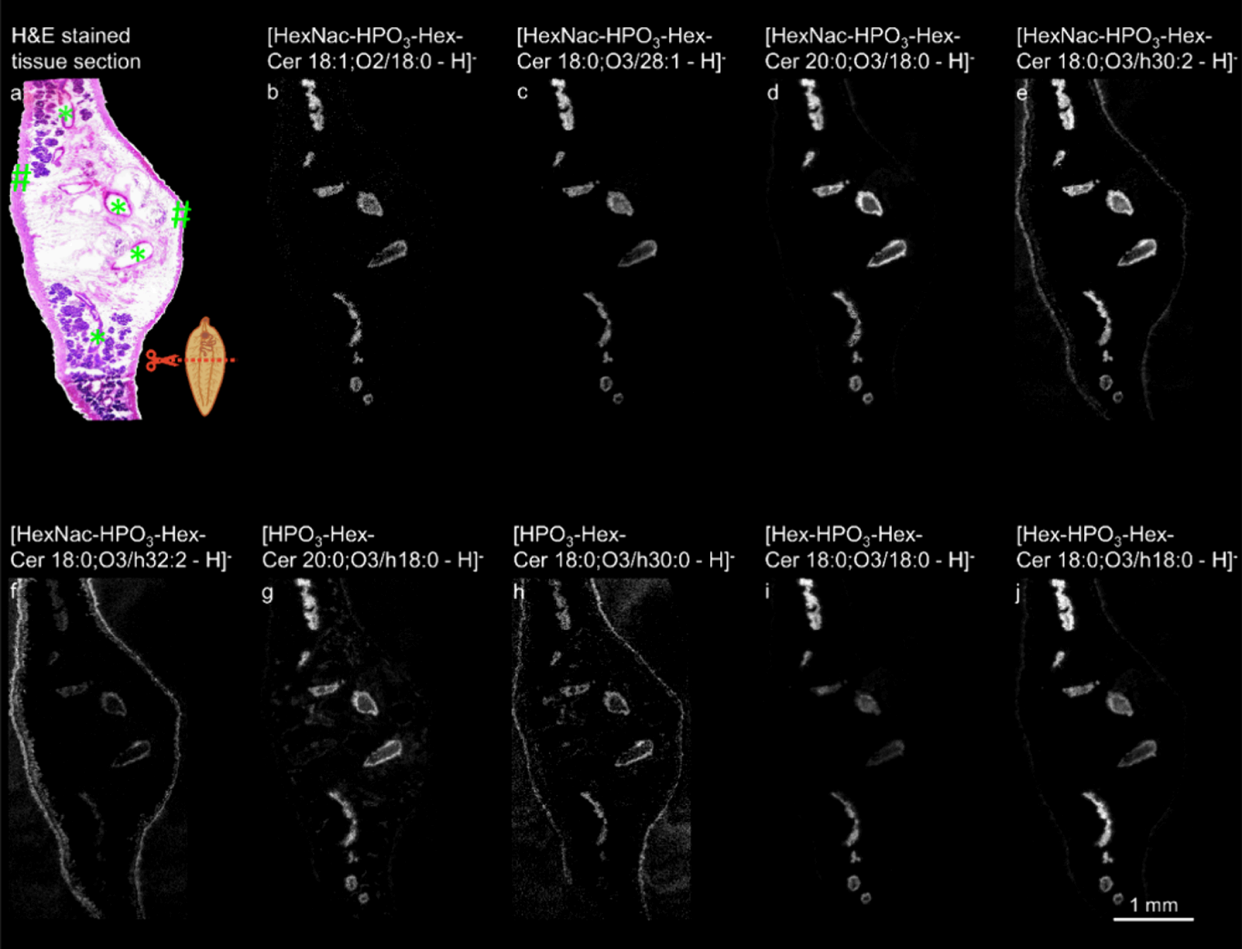
Spatial distributions of phosphate-containing glycosphingolipids in a cross section of adult *F. hepatica*. (a) Hematoxylin and eosin (H&E) staining of an *F. hepatica*transversal section after AP-SMALDI MSI measurement. The *F. hepatica* pictogram illustrates the transverse sectional plane. Intestines are signed with asterisks and the tegument with hashes. (b) Single-ion image of HexNac-HPO_3_-Hex-Cer 18:1;O2/18:0 ([M-H]^−^ at *m*/*z* 1009.6349). (c) Single-ion image of HexNac-HPO_3_-Hex-Cer 18:0;O3/28:1 ([M-H]^−^ at *m*/*z* 1165.7861). (d) Single-ion image of HexNac-HPO_3_-Hex-Cer 20:0;O3/18:0 ([M-H]^−^ at *m*/*z* 1055.6769). (e) Single-ion image of HexNac-HPO_3_-Hex-Cer 18:0;O3/h30:2 ([M-H]^−^ at *m*/*z* 1207.8279). (f) Single-ion image of HexNac-HPO_3_-Hex-Cer 18:0;O3/h32:2 ([M-H]^−^ at *m*/*z* 1235.8279). (g) Single-ion image of HPO_3_-Hex-Cer-20:0;O3/h18:0 ([M-H]^−^ at *m*/*z* 868.5921). (h) Single-ion image of HPO_3_-Hex-Cer-18:0;O3/h30:0 ([M-H]^−^ at *m*/*z* 1008.7822). (i) Single-ion image of Hex-HPO_3_-Hex-Cer 18:0;O3/18:0 ([M-H]^−^ at *m*/*z* 986.6187). (j) Single-ion image of Hex-HPO_3_-Hex-Cer 18:0;O3/h18:0 ([M-H]^−^ at *m*/*z* 1002.6136). The AP-SMALDI MSI measurement was performed with a 10 μm step size.

To spatially map these GSLs within the tissue, we employed AP-SMALDI MSI experiments in negative-ion mode, making use of the GSLs identified by nano-HILIC MS/MS. This approach was sensitive enough to facilitate the visualization of 30 out of the 35 phosphate-containing GSLs. For the remaining five GSL species, the abundance was too low to image the ion signal of these species. Figure 3 illustrates representative examples, emphasizing diverse compositions of GSLs showing different distribution patterns.

Three GSLs (saccharide composition HexNac-HPO_3_-Hex) associated with the ceramide composition Cer 18:1;O2/18:0 ([M-H]^−^ at *m*/*z* 1009.6349), Cer 18:1;O3/28:1 ([M-H]^−^ at *m*/*z* 1165.7861), and Cer 20:0;O3/18:0 ([M-H]^−^ at *m*/*z* 1055.6769) were predominately found concentrated across the intestine (Figure 3b–d). In contrast, the species HexNac-HPO_3_-Hex-Cer 18:0;O3/h30:2 ([M-H]^−^ at *m*/*z* 1207.8279 and HexNac-HPO_3_-Hex-Cer 18:0;O3/h32:2 ([M-H]^−^ at *m*/*z* 1235.8279) exhibited a distribution encompassing both the intestine and tegument of *F. hepatica* (Figure 3e,f). GSLs HPO_3_-Hex-Cer-20:0;O3/h18:0 ([M-H]^−^ at *m*/*z* 868.5921) and HPO_3_-Hex-Cer-18:0;O3/h30:0 ([M-H]^−^ at *m*/*z* 1008.7822) had distribution patterns similar to the previously mentioned two species (Figure 3g,h). Regarding the new head group composition Hex-HPO_3_-Hex, two GSLs, Hex-HPO_3_-Hex-Cer 18:0;O3/18:0 ([M-H]^−^ at *m*/*z* 986.6187) and Hex-HPO_3_-Hex-Cer 18:0;O3/h18:0 ([M-H]^−^ at *m*/*z* 1002.6136), were identified. Their spatial distributions are depicted in Figure 3i,j. Both species showed a prominent accumulation in the intestine, and the latter was also detected in the tegument.

These findings underscore the importance of assessing GSL distributions at a molecularly intact level without ceramide moiety cleavage, different from common practice in a previous study.^24^ Our results suggest that the phosphate-containing GSLs can be categorized into two groups, based on the contained fatty acids (FAs)—hydroxylated and nonhydroxylated FAs. While the former group exhibited a distribution spanning both the intestine and tegument, the latter was predominantly found in the intestine. This indicates a potential relevance of the hydroxylated fatty acid for the host–parasite interaction. The location of nonhydroxylated fatty acids of phosphate-containing GSLs may reflect the specific lipid metabolism of the parasite, including the modification and utilization of FAs in its intestine. A study by Wuhrer and co-workers reported that trihexosylceramides of *Fasciola gigantica* (*F. gigantica*), containing dihydroxylated ceramides, exhibited the strongest recognition to the Shiga toxin B1 subunit, which is known to bind to Gb_3_ species. In contrast, the Shiga toxin B1 subunit only exhibited a weak binding to trihexosylceramides with tri- and tetrahydroxylated ceramides of *F. gigantica*, which were dominant in their LC fractions.^16^ This supports our hypothesis that the ceramide moiety potentially plays a role in GSL function.

Moreover, we identified very long-chain fatty acids (VLCFA), with up to 32 carbon atoms incorporated into phosphate-containing GSLs. Next, we conducted AP-SMALDI MSI experiments in positive-ion mode with adult *F*. *hepatica,* to determine whether the spatial distributions of neutral GSLs exhibit a similar ceramide dependence. In general, we noted a predominant distribution of mono- or trihexoses across the tegument and intestine. Among the visualized compounds, GSL species with a VLCFA as part of the ceramide were found accumulated in the tegument, as exemplarily shown in Figure S3. While the function of these atypical FAs in *F. hepatica* is unknown, we may learn from their role in mammals. For example, ceramides with VLCFA are known to be important for the permeability integrity of the epidermis in mammals, with disorders leading to skin barrier defects.^25,26^ Therefore, incorporating GSL with different ceramides into the glycocalyx might represent a protective mechanism for the parasite.

As *F. hepatica* is not capable of *de novo* FA synthesis, these VLCFA must either be taken up from the host, or produced by the parasite via FA elongation.^27^ We were able to identify four elongase genes in the *F. hepatica* genome (D915_006030, D915_002071, D915_002072, and D915_004203), which supports the mechanism of chain elongation by the parasite. The tissue types showing expression of three of these genes were identified with the help of our spatial transcriptome of adult *F. hepatica* (Figure S4a).^28^ The elongases D915_006030 and D915_002072 were detected across several different tissue types with the strongest expression in the tegument, as shown in Figure S4b. Future knockdown or inhibitor experiments might reveal if there is a direct connection between the expression of these genes and the presence of the VLCFA in GSLs.

The curated GSL database for adult *F. hepatica* was subsequently utilized to investigate the local distributions of GSLs of migrating *F. hepatica* in the liver parenchyma, which cause most of the pathogenesis.

### AP-SMALDI MSI Reveals the Presence of Plasmalogen Phospholipids at the Host–Parasite Interface

**Figure 4:**
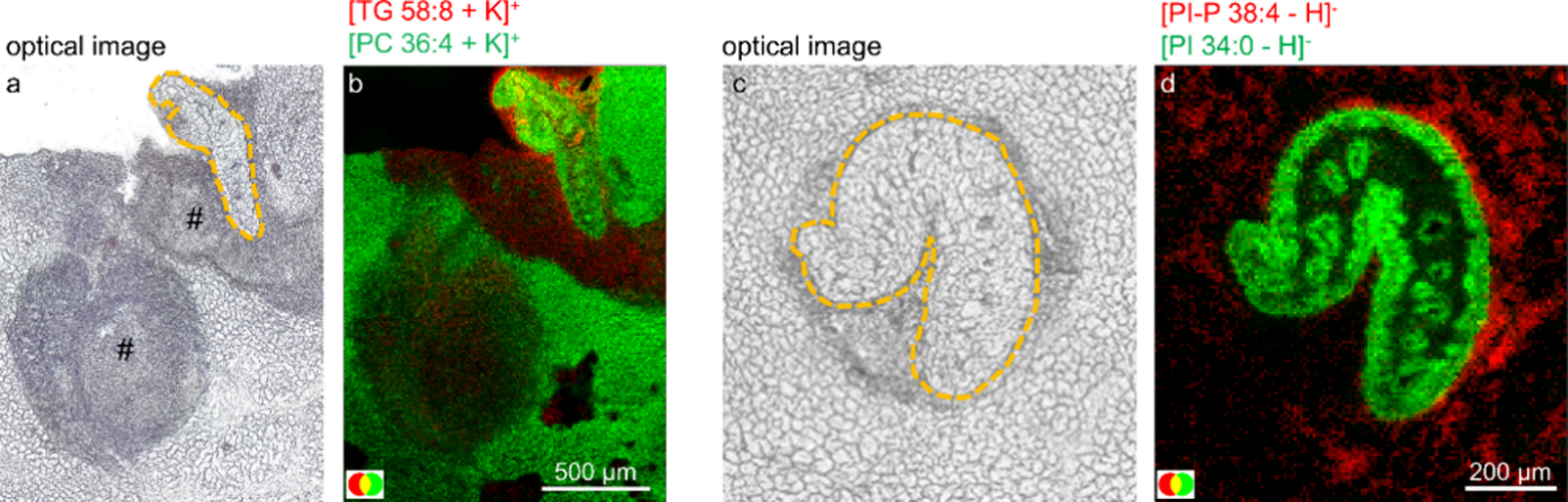
Lipidomics analysis of the rat liver infected with *F. hepatica*at the acute infection stage. (a) Optical image before AP-SMALDI MSI analysis of a rat liver tissue section during the acute infection stage. The outline of the migrating *F. hepatica*is emphasized with an orange dotted line, and tissue lesions are highlighted by a hash. (b) Positive-ion RG-overlay image corresponding to the optical image (a), showing TG 58:8 ([M+K]^+^ at *m*/*z* 969.7308) in red and PC 36:4 ([M+K]^+^ at *m*/*z* 820.5253) in green. (c) Optical image before AP-SMALDI MSI analysis of a rat liver tissue section during the acute infection stage. The outline of the migrating *F. hepatica*is emphasized with an orange dotted line. (d) Negative-ion RG-overlay image corresponding to (h), showing PI–P 38:4 ([M-H]^−^ at *m*/*z* 869.5549) in red and PI 34:0 ([M-H]^−^ at *m*/*z* 837.5499) in green. AP-SMALDI MSI measurements in this figure were performed with a 7 μm step size.

To study acute infection and effects of parasite migration, tissue sections of the rat liver harboring migrating *F. hepatica* (Figure 4a,c) were evaluated on the lipid level first before investigating GSL distributions. Lipids are well-known indicators for parasite and tissue anatomy and molecular interactions and are easily accessible in AP-SMALDI MSI experiments.^29−31^ As illustrated in the MS image in Figure 4b in red, the triacylglyceride (TG) 58:8 ([M+K]^+^ at *m*/*z* 969.7308) exemplifies an upregulated lipid, appearing more abundant in areas of tissue lesions compared to healthy rat liver controls (Figure S5c). This observed trend extended across various species annotated as TGs, as illustrated in Figure S6. This aligns with our supporting semiquantitative RP-LC MS/MS data, which are described in Supplementary Note 8, indicating an upregulation of TGs in acutely infected rat livers (Figure S7). This observation is congruent with the literature, associating TGs with energy supply during tissue repair.^32^ Additionally, TGs seem to be higher in abundance in the tissue lesion surrounding the migrating *F. hepatica* compared to the other tissue lesion. This accumulation of TGs can potentially be explained with *F. hepatica* inducing oxidative stress,^33^ which in general can lead to the accumulation of triglycerides.^34^

In contrast, phosphatidylcholine (PC) 36:4 ([M+K]^+^ at *m*/*z* 820.5253) shown in Figure 4b in green exhibits decreased abundance in the regions impacted by tissue lesions compared to the surrounding healthy tissue but was found to be homogeneously distributed in the liver tissue of healthy control rats (Figure S5b). Here, our supporting RP-LC MS/MS data again align with this observation, with several phospholipids being significantly decreased in the rat liver in the acute infection stage compared to the control (Figure S7). The observed decrease in phospholipids concurs with the established enzymatic activity of *F*. *hepatica:* phospholipases in the tegument, vomitus, or secretions produce free FAs.^6,7^ As the parasite cannot synthesize fatty acids *de novo*,^35^ degrading phospholipids in the liver tissue can prove beneficial. By this, the amount of small building blocks, which are taken up through the tegument, like free FAs as well as phospholipid headgroups such as choline, can be increased.

Beyond the unique distribution of lipids in healthy hepatic tissues or lesions, a subset of lipids exhibited elevated abundances at the host–parasite interface. For example, PI–P 38:4 ([M-H]^−^ at *m*/*z* 869.5549) in Figure 4d in red showed an accumulation in tissues directly adjacent to the parasite but had low pixel coverage in the control sample as shown in Figure S8. The accumulation of several other plasmalogen phospholipids, highlighted in Figure S9, at the host–parasite interface raises the question of their potential association with immune cells of the host as levels of plasmalogen species are usually low in the liver.^36^ Potentially, plasmalogen species could serve as markers for macrophages. These types of immune cells are highly relevant in regulating the immune response during fascioliasis, and they are known to contain several plasmalogen species.^37,38^ A similar distribution to that for PI–P 38:4 was also found for the annotation bis(monoacylglycero)phosphate (BMP) 34:0, shown in Figure S10b. This supports our hypothesis of plasmalogens as immune cell markers in fascioliasis infection, as it has already been shown in the mouse brain tissue that BMP species can serve as macrophage markers.^39^ However, to establish a firm link between the two studies, it is crucial for our data set to confirm BMP 34:0 by MS/MS experiments as BMPs are structural isomers to phosphatidylglycerols. In addition, immunohistochemical experiments will also prove beneficial in future studies.

Furthermore, we found distributions of lipid species specific for migrating *F. hepatica*. For example, PI 34:0 ([M-H]^−^ at *m*/*z* 837.5499) is shown in Figure 4d in green, which was neither detected during our AP-SMALDI MSI analysis in hepatic tissues nor was it identified during RP-LC MS/MS data analysis. Although not capable of *de novo* fatty acid synthesis, it is known that *Fasciola* spp. can modify fatty acids.^27^

#### AP-SMALDI MSI of GSLs in Immature Migrating *F. hepatica* Indicates Tegument Remodeling

**Figure 5:**
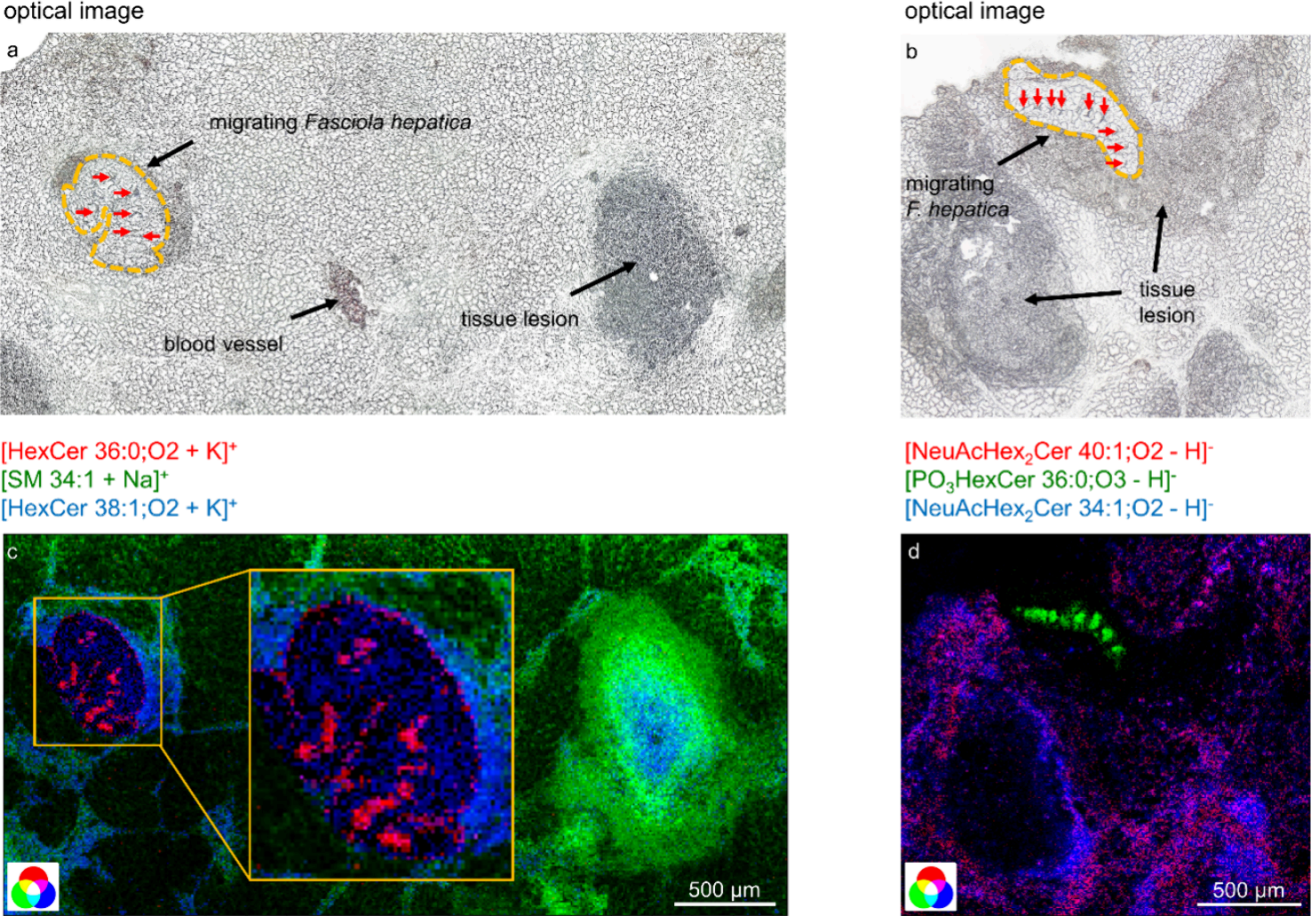
Glycosphingolipids characteristic of the rat liver infected with *F. hepatica*at the acute infection stage. (a,b) Optical images of liver tissue sections of rats during the acute infection stage. The outlines of the migrating parasites are emphasized with an orange dotted line, and the intestine is indicated by red arrows. (c) Positive-ion AP-SMALDI RGB-overlay image corresponding to (a) Hex-Cer 36:0;O2 ([M+K]^+^ at *m*/*z* 768.5750) in red, SM 34:1 ([M + Na]^+^*m*/*z* 725.5568) in green, and Hex-Cer 38:1;O2 ([M+K]^+^*m*/*z* 794.5907) in blue. The measurement was performed with a 10 μm step size. (d) Negative-ion AP-SMALDI RGB-overlay image corresponding to (b), showing NeuAcHex_2_Cer 40:1;O2 ([M-H]^−^ at *m*/*z* 1235.8002) in red, HPO_3_-Hex-Cer 36:0;O3 ([M-H]^−^ at *m*/*z* 824.5661) in green, and NeuAcHex_2_Cer 34:1;O2 ([M-H]^−^ at *m*/*z* 1151.7063) in blue. The measurement was performed with a 7 μm step size.

To study the effects of parasite migration in host tissues on a higher systemic level, rat liver tissue sections containing immature *F. hepatica* (Figure 5a,b) were investigated by imaging the GSL species expected from the nano-HILIC MS/MS results for isolated adult *F. hepatica*. By performing AP-SMALDI MSI experiments in positive- and negative-ion modes, we were able to detect GSL species that were only present in immature *F. hepatica* and not in the liver tissue. As an example for the positive-ion mode, Figure 5c showcases Hex-Cer 36:0;O2 ([M+K]^+^ at *m*/*z* 768.5750) as a representative GSL, highlighted in red. For the negative-ion mode, HPO_3_-Hex-Cer 36:0;O3 ([M-H]^−^ at *m*/*z* 824.5661) is shown in Figure 5d in green. Both examples are well-suited to showcase the benefits of a spatial resolution below 20 μm pixel size in order to resolve small histological features in the tissue sections such as the tegument (10–15 μm) and the intestine (10–60 μm) of the migrating parasite. Here, Hex-Cer 36:0;O2 was found to be distributed in the tegument and intestine of the migrating parasite and HPO_3_-Hex-Cer 36:0;O3 in the intestine.

Previous investigations have explored various glycoconjugates, such as like N-glycans, during the migratory stages, and a reduction of high mannose and fucose-containing glycans upon an earlier migration phase of the worms across the jejunum.^40,41^ However, to the best of our knowledge, the role of GSLs within the migratory stage has not been the subject of studies so far. Therefore, with our AP-SMALDI MSI data, we provide for the first time insights into the distribution of GSLs of migrating immature parasites through liver parenchyma and contribute to a better understanding of this class of molecules.

Simple hexosylceramides specific for immature *F. hepatica* (Figure 5c) and also trihexosylceramide species, distributed across the tegument (Figure S11), were detected. The detection of mainly mono- or trihexosylceramide species is in line with our previous observation on GSLs in adult *F*. *hepatica.* Unlike specific monohexosylceramides, trihexosylceramides were not detected in the intestine. This disparity suggests a potential mechanism wherein monohexosyl GSLs are assimilated from the host through feeding via the oral sucker (scavenge nutrients from their hosts), subsequently undergoing metabolism to yield more parasite-specific GSLs. This hypothesis is supported by the fact that larger molecules are acquired by the parasite from the host by the oral sucker and not via the tegument. The tegument, on the other hand, is responsible for the uptake of low-molecular-weight molecules like FAs, amino acids, and monosaccharides.^42^ Further experiments are required to validate the significance of these GSLs, distributed across the tegument, for liver migration.

**Figure 6:**
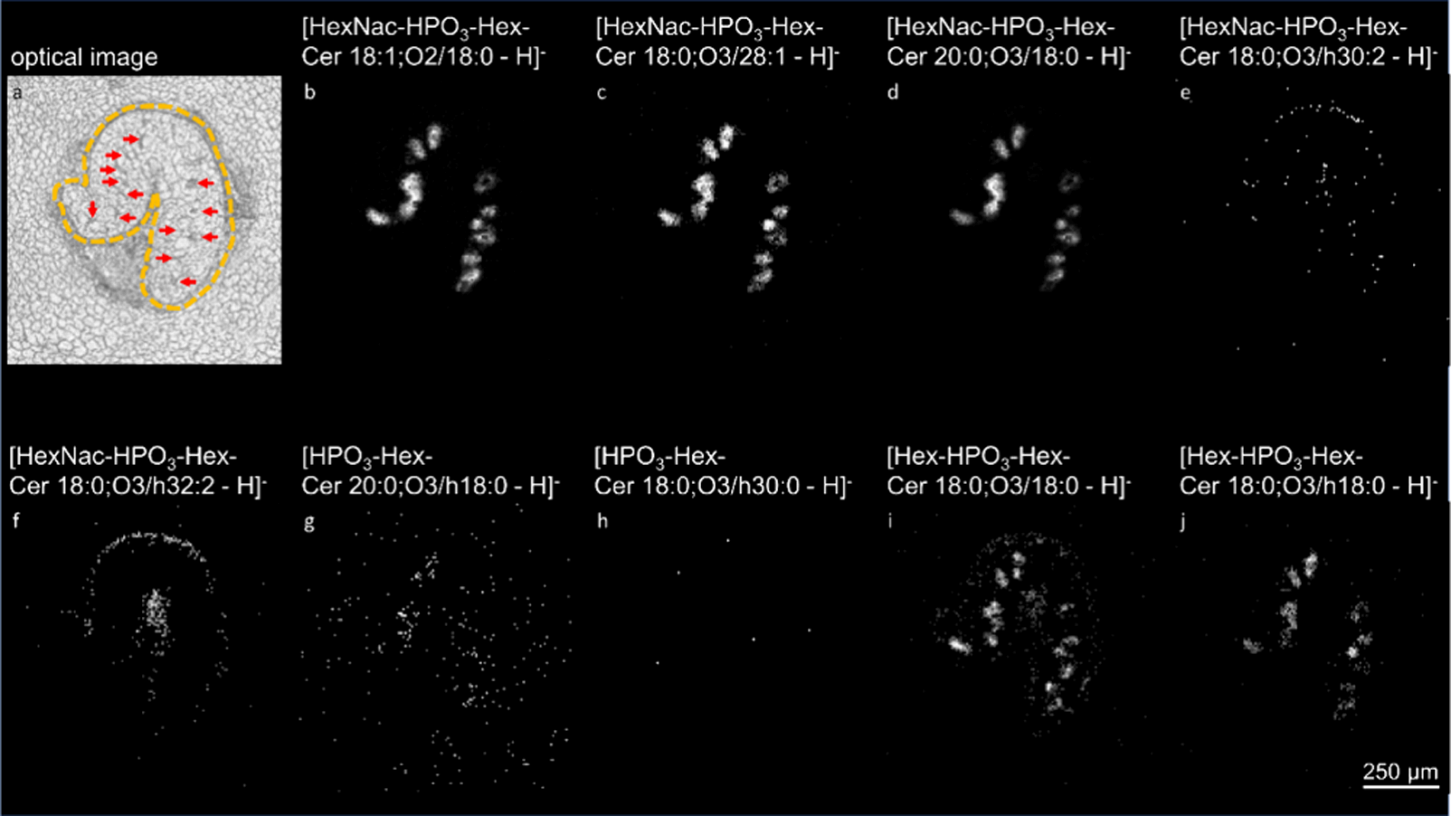
Spatial distribution of phosphate-containing glycosphingolipids in immature *F. hepatica*. (a) Optical image of a section of an immature *F. hepatica*, emphasized by an orange dotted line, surrounded by a hepatic tissue. The intestine of the parasite is highlighted by red arrows. (b) Single-ion image of HexNac-HPO_3_-Hex-Cer 18:1;O2/18:0 ([M-H]^−^ at *m*/*z* 1009.6349). (c) Single-ion image of HexNac-HPO_3_-Hex-Cer 18:0;O3/28:1 ([M-H]^−^ at *m*/*z* 1165.7861). (d) Single-ion image of HexNac-HPO_3_-Hex-Cer 20:0;O3/18:0 ([M-H]^−^ at *m*/*z* 1055.6769). (e) Single-ion image of HexNac-HPO_3_-Hex-Cer 18:0;O3/h30:2 ([M-H]^−^ at *m*/*z* 1207.8279). (f) Single-ion image of HexNac-HPO_3_-Hex-Cer 18:0;O3/h32:2 ([M-H]^−^ at *m*/*z* 1235.8279). (g) Single-ion image of HPO_3_-Hex-Cer-20:0;O3/h18:0 ([M-H]^−^ at *m*/*z* 868.5921). (h) Single-ion image of HPO_3_-Hex-Cer-18:0;O3/h30:0 ([M-H]^−^ at *m*/*z* 1008.7822. (i) Single-ion image of Hex-HPO_3_-Hex-Cer 18:0;O3/18:0 ([M-H]^−^ at *m*/*z* 986.6187). (j) Single-ion image of Hex-HPO_3_-Hex-Cer 18:0;O3/h18:0 ([M-H]^−^ at *m*/*z* 1002.6136). The AP-SMALDI MSI measurement was performed with a 7 μm step size.

The distribution patterns of specific phosphate-containing GSL species without hydroxylated FAs as part of their ceramides were similar in migrating liver flukes compared to adult parasites. We successfully detected these GSLs in migrating *F. hepatica* across the intestine. They appeared comparable in abundance to the levels found in the adult *F*. *hepatica.*Figure 6 shows the same GSL species for immature *F. hepatica* as discussed above for adult *F. hepatica*, which are HexNac-HOP_3_-Hex-Cer 18:1;O2/18:0, HexNac-HOP_3_-Hex-Cer 18:1;O2/18:0, HexNac-HOP_3_-Hex-Cer 18:1;O2/18:0, HexNac-HOP_3_-Hex-Cer 18:1;O2/18:0, and HexNac-HOP_3_-Hex-Cer 18:1;O2/18:0 (Figure 6b–d,i,j). However, there are also clear differences between adult and immature *F. hepatica*, when looking at GSL species with a hydroxylated FA as part of the ceramide moiety. These species were hardly detectable in the migrating *F. hepatica*. As examples, the GSL species HexNac-HOP_3_-Hex-Cer 18:1;O2/18:0, HexNac-HOP_3_-Hex-Cer 18:1;O2/18:0, HexNac-HOP_3_-Hex-Cer 18:1;O2/18:0, and HexNac-HOP_3_-Hex-Cer 18:1;O2/18:0 are shown in Figure 6e–h. Each of these species in immature *F. hepatica* was found less accumulated in the intestine or tegument compared to the results for adult *F. hepatica*. An exception was the GSL Hex-HPO_3_-Hex-Cer 18:0;O3/h18:0, which was found to be distributed across the intestine. These results raise the question of whether this is an example for tegumental remodeling, a well-known phenomenon in *F. hepatica*.^43^ There is a chance that phosphate-containing GSLs are more relevant for tegumental function in the adult life stage of *F. hepatica*, with several species found to be distributed in the tegument in direct contact with the host. However, it should be considered that there are possible *in vivo* and *in vitro* differences between the two parasite stages that could have an influence on the GSL distribution. However, we believe that this effect does not have a major impact because the GSL profile of adult *F. hepatica* is close to previous studies as described earlier. In addition, the existing overlap in both parasite stages of some of the identified GSL species suggests that they are robust against different methods of treatment. Therefore, we favor the conclusion that the differences are not due to differences in handling but to age dependence and may be relevant to natural infection.

Another phenomenon is illustrated by the ion image of HexNac-HPO_3_-Hex-Cer 18:1;O2/18:0, maximally brightened in Figure S12. It shows HexNac-HPO_3_-Hex-Cer 18:1;O2/18:0 mainly detected across the entire immature *F. hepatica* but with low signal intensities also in hepatic tissue lesions. This led us to the hypothesis that these specific GSLs might be excreted during the migration process and might be part of the host–parasite interaction during the migratory phase.

Besides parasite-typical GSL species, profiling of host GSLs can also be valuable to better understand the interaction of the host and the parasite and in particular the host’s immune response. GSL species are generally known to be involved in immune responses.^44^ Therefore, we additionally profiled GSL species of the liver and bile duct samples at the acute and chronic infection stage.

#### Comprehensive GSL Profiling Unveils Elevated Levels in Rat Livers during Acute Infection

After nano-HILIC MS/MS analysis, we classified the identified GSL species into five different groups, including mono/di/trihexossylceramides, complex neutral GSLs with at least four saccharide units, neutral GSLs containing at least one fucose moiety, and acidic GSLs and GSLs with a phosphate group (see Figure S13). Regarding the mono/di/trihexoses, we identified a higher number of species for the acute infection stage (11) and the bile duct (18) compared to the chronic infection (6) and the controls for the acute infection (4) and chronic infection (7). For the more complex GSLs with at least four saccharide units, the bile duct showed an increased number of identifications (12) compared to all liver samples. Neutral GSLs containing a fucose saccharide were higher in number for the bile duct (9) but also for the liver samples from the acute infection stage (8), compared to the other three groups. The number of identified acidic GSLs was the highest across all samples. However, we could not observe a specific trend. For the rat liver of the acute infection stage, we identified 41 and for the corresponding control 38 acidic GSL species, respectively. A similar number of identifications was observed for the rat liver of the chronic infection stage (40) compared to their control (37). Only the bile duct sample showed a larger difference with 50 identified acidic GSLs. The identification numbers for GSLs containing a phosphate group were small across all groups, with 4 identifications for the acute infection, 1 for the corresponding control, 2 for the chronic infection, 6 for the corresponding control, and 4 for the bile duct.

**Figure 7:**
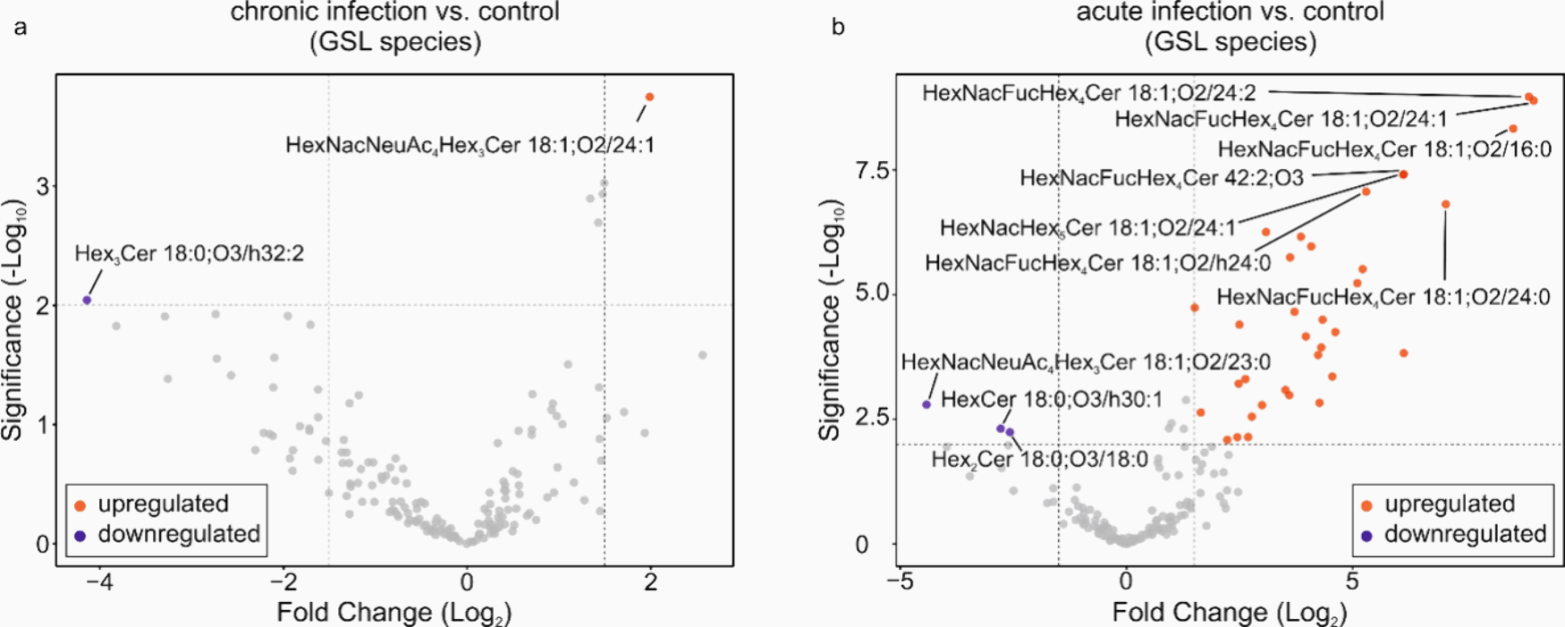
(a) Volcano plot comparing the results from MS-DIAL data analysis for GSLs after normalization. Livers from rats at the chronic infection stage were compared to healthy control livers. Red circles correspond to an increased species and a blue circle to decreased species. (b) Volcano plot comparing the results from MS-DIAL data analysis for GSLs after normalization. Livers from rats at the acute infection stage were compared to healthy control livers. Red circles correspond to increased species and blue circles to decreased species. The fold-change threshold was set to −1.5 to 1.5, and the significance threshold was set to *p* < 0.01 for both plots.

Through the integration of an internal standard into our nano-HILIC MS methodology, we enabled the adjustment of signal intensities but also contributed to the enhancement of data accuracy and reliability. Principal component analysis (PCA) based on GSLs delineated all examined rat liver and bile duct tissue samples (Figure S14). Notably, the acute infection stage demonstrates a separation from the chronic infection stage and both control groups. As the liver tissue of chronically infected animals in this study did not show gross pathological lesions (Figure S15), it can be assumed that liver regeneration had already taken place, so that the tissue resembled a healthy tissue again. Indeed, the volcano plot shown in Figure 7a indicates a normalization in GSL levels, although this molecule class is potentially relevant during infection. In the plot, only minor differences are apparent for the comparison of the GSLs from the liver of the chronic infection stage to the control. In contrast, we detected increased levels of GSLs in the livers of rats undergoing acute infection compared to healthy controls (Figure 7b). In particular, fucose-containing GSLs showed significant upregulation for the acute infection compared to the control. These results support the conjecture of GSL participation in infection-related immune responses. In comparison to the adult *F. hepatica*, the heat map shown in Figure S16 clearly highlights the differences between parasite- and tissue-specific GSLs, with many GSL species being specific for the parasite or the host.

### AP-SMALDI MSI of the Rat Liver at the Acute Infection Stage to Study Host–Parasite Interactions

Understanding the spatial distribution of GSLs within tissues is helpful for identifying those species potentially involved in direct host–parasite interactions. Here, the tissue sections containing the immature *F. hepatica* but also areas of tissue lesions were re-evaluated (Figure 5a,b). For annotations of GSLs, we integrated our curated GSL database into METASPACE. For that, we combined the GSL identifications of host tissue samples as well as isolated adult *F. hepatica*. For the AP-SMALDI MSI measurements, distinct distribution patterns were discernible for neutral GSL species. Some monohexosylceramides were characteristic of both *F. hepatica* and the surrounding liver tissue. The GSL Hex-Cer 38:1;O2 ([M+K]^+^*m*/*z* 794.5907) in Figure 5c in blue is given as an example. The species appeared to be accumulated at the host–parasite interface and the center of the tissue lesion. Here, the question arises if the parasite eventually sheds these GSLs to slow down liver regeneration. A previous study already showed that glucosylceramides impaired liver regeneration, which led to our hypothesis.^45^ Alternatively, different GSLs may be associated with the presence of certain immune cells, as we suggested in our previous study for the hamster liver.^23^ Because Hex-Cer 38:1;O2 is not exclusively distributed at the host–parasite interface, this species could also serve as a marker for specific immune cells. Eosinophils, for example, are known to limit tissue damage induced by *F. hepatica*.^46^ Moreover, eosinophils are known to promote liver regeneration.^47^

Additionally, the distribution of SM 34:1 ([M + Na]^+^*m*/*z* 725.5568) is shown in Figure 5c in green. This species showed an accumulation within the tissue lesion, with less accumulation in the center of the lesion. Other distributions of SM species are shown in Figure S17. As SMs and GSLs both belong to the class of sphingolipids, investigating SMs in addition to GSLs can help to reveal metabolic processes. Therefore, it is beneficial that we also detect SM species during our nano-HILIC MS/MS analyses. Regarding more complex GSLs, we were not able to visualize fucose-containing GSLs in the liver of the acutely infected rats, although identified as a significant upregulated group of GSL species through our nano-HILIC MS/MS data analysis. Nevertheless, we were able to visualize fucose-containing GSL species in bile duct tissue sections, as exemplarily shown in Figure S18. However, the lack of local information in rat liver tissue sections from acute infection makes it difficult to interpret the possible role of fucose-containing GSL species. Improving the detection of glycosphingolipids during MALDI MSI analysis and thus revealing their distributions in the tissue may help to narrow down a possible role in biological processes in the future.

In the case of acidic GSLs, we mainly observed the distributions of NeuAcHex_2_Cer species. Among these, we found similar distribution patterns. As depicted in the RGB-overlay image in Figure 5d, NeuAcHex_2_Cer 34:1;O2 ([M-H]^−^ at *m*/*z* 1151.7063) shown in blue has a similar distribution compared to NeuAcHex_2_Cer 40:1;O2 ([M-H]^−^ at *m*/*z* 1235.8002) shown in red. The results align with a study demonstrating an increase in ganglioside production during liver regeneration several days after partial hepatectomy.^48^ The AP-SMALDI MSI data for NeuAcHex_2_Cer signals are furthermore backed by the semiquantitative nano-HILIC MS/MS data. Here, 13 out of 19 compounds included in our database showed a significant (*p* < 0.05) upregulation in the rat liver of the acute infection stage compared to the control.

## Conclusions

Our previously reported method for studying GSLs has proven applicable for parasite samples. We identified the spatial distribution of GSLs in isolated adult *F. hepatica*. We were able to identify GSLs with VLCFA as part of the tegument and revealed a local overlap with the host’s own elongase enzymes. As the parasite may actively synthesize these GSLs with VLCFA, this could indicate a protective function for the parasite and thus represent a feasible drug target. The spatially resolved AP-SMALDI analysis of GSLs also provides indications about the metabolism. Here, we may ascertain that *F. hepatica* converts hexosylceramides to trihexosylceramides. Because these species are mainly distributed in the tegument, they may also be an important component in the host–parasite interaction, with the function still to be determined. Additionally, we presented the first identification of GSL species in the migratory stage, including the visualization of the distributions of phosphate-containing glycosphingolipids specific for the liver fluke *F. hepatica*. Here, the differences from adult *F. hepatica* indicate that we can elucidate tegument remodeling. In particular, a hydroxyl group in the FAs of the GSL of adult *F. hepatica* appears to be a major difference between the two growth stages. Because for adult *F. hepatica*, the hydroxylated GSLs are located within the tegument, they could be crucial for the host–parasite interaction at this stage. Thus, this structural feature at the molecular level could be targeted in future drug development studies. GSL profiling of different infection stages additionally revealed elevated levels of GSLs, especially for tissues in direct contact with the parasite: the rat liver during the acute infection stage and the bile duct during the chronic infection stage. Furthermore, we employed high-resolution AP-SMALDI MSI for the first time to scrutinize the interaction of migrating *F. hepatica* with the surrounding liver tissue. This enabled us to identify potential immune cell markers. The distribution of some hexosylceramides might be characteristic of eosinophils. In addition to GSLs, there were also distinctive characteristics among other lipid classes. In particular, plasmalogenic phospholipids showed an accumulation at the host–parasite interface and could serve as immune cell markers for macrophages. Therefore, future more detailed studies on other lipid classes seem equally useful to better understand the complex interplay between the host and the parasite. In this context, it will also be important in the future to determine whether the findings obtained for our rat model can be transferred to human infections. Overall, besides the phospholipids, our data identified further different GSL species such as fucose-containing GSLs, phosphate-containing GSLs, or simple hexosylceramides in the tissue, which are possibly involved in the host–parasite interaction and form the basis for further investigations.

## Materials and Methods

### Chemicals

Acetonitrile, isopropanol, methanol, water (HiPerSolv), and gelatin powder were purchased from VWR International GmbH (Darmstadt, Germany). Ammonium acetate (AmAc) and 2,5-dihydroxyacetophenone (DHAP) were purchased from Alfa Aesar (Kandel, Germany). Formic acid (FA), methyl-*tert*-butylether (MTBE), hematoxylin, eosin Y, and Eukitt were purchased from Sigma-Aldrich (Darmstadt, Germany). Chloroform and sodium hydroxide were purchased from Carl Roth GmbH + Co. KG (Kalsruhe, Germany), and ammonium formate (AmF) was purchased from Acros Organics (Geel, Belgium). Ethanol, isopentane, xylene, and glacial acetic acid were purchased from Merck (Darmstadt, Germany).

### Tissue and Sample Preparation

#### Ethical Statement

Animal experiments using rats (*Rattus norvegicus*) as model hosts were performed in accordance with Directive 2010/63/EU on the protection of animals used for scientific purposes and the German Animal Welfare Act. The experiments were approved by the Regional Council (Regierungspraesidium) Giessen (V54-19c20 15 h 02 GI 18/10 Nr. A16/2018).

#### Animal Experiments

Male Wistar rats RjHan:WI (*Rattus norvegicus*, Janvier, France) were used as model hosts for *F. hepatica* infection. Animals (5–7-weeks-old) were orally infected with 25 metacercariae of an Italian parasite strain (Ridgeway Research, UK) or kept as uninfected controls. Infected and control animals were kept in the same facility and were housed under controlled conditions (light cycle, temperature, humidity, water, and food).

#### Harvesting of *F. hepatica*

Adult flukes were collected from the common bile duct at 14 and 20 weeks p.i. Worms were kept in RPMI 1640 (Gibco, Thermo Fisher Scientific, Germany) supplemented with a 5% chicken serum (Gibco, Thermo Fisher Scientific) and 1% ABAM solution (c.c.pro, Germany) at 37 °C and 5% CO_2_ overnight to allow clearance of gut contents. Parasites were then flash frozen in liquid nitrogen for lipid extraction or embedded in 8 wt % aqueous gelatin solution and frozen on dry ice for AP-SMALDI MSI analyses. Worm samples were stored at −80 °C until further use.

#### Tissue Sampling

Liver samples were collected from *F. hepatica*-infected animals as well as noninfected controls at 4 and 14 weeks p.i. (9–11 and 20–21 weeks animal age). Samples were all taken from the right median lobe, where yellowish tortuous tracts were visible in the parenchyma of animals infected with immature worms (4 weeks p.i.). From animals infected with adult worms (14 weeks p.i.), we additionally sampled the dilated and thickened proximal part of the common bile duct, after removal of the worms. Tissue samples were frozen in isopentane prechilled on dry ice and stored at −80 °C until further use.

#### AP-SMALDI MSI

To conduct AP-SMALDI MSI measurements, we subjected the samples to a cryochamber at −20 °C for 30 min. *F. hepatica* worms, embedded in gelatin, were sliced into 20 μm-thick transversal sections using a cryotome (HM525 cryostat, Thermo Fisher Scientific, Bremen, Germany). Care was taken to ensure that the tissue sections included intestines as well as the tegument as histological features, while other organs of *F. hepatica* were optional. Rat liver and bile duct tissues underwent the same cutting process but without prior embedding. The sections were thaw-mounted onto microscopic glass slides, and microscopic images of them were recorded (VHX-5000; Keyence, Osaka, Japan) before matrix application. Tissue sections were stored at −80 °C until further use. Prior to AP-SMALDI measurements, tissue sections were thawed for 20 min in a desiccator at room temperature. The matrix 2,5-dihydroxyacetophenone (DHAP) was applied by sublimation as described previously.^23^

All MSI experiments were performed using a high-resolution atmospheric-pressure autofocusing scanning microprobe MALDI imaging ion source (AP-SMALDI^5^ AF, TransMIT GmbH, Giessen, Germany)^21^ coupled to an orbital trapping mass spectrometer (Thermo Scientific Q Exactive HF, Thermo Fisher Scientific, Bremen, Germany). More details are described in the Supplementary Note 1.

AP-SMALDI MSI data underlying this study are openly available in the METASPACE database at https://metaspace2020.eu/project/GSL_and_LIPIDS_Fhepatica_and_Inf. For GSL annotation, we uploaded a GSL database to METASPACE, which was based on the nano-HILIC MS/MS experiments described in the upcoming section.

#### LC MS/MS

For LC MS/MS, tissue samples were homogenized by using a bead mill (Mini-Mill PULVERISETTE 23, Fritsch, Idar-Oberstein, Germany). Prior to homogenization, 50 μL of AmAc solution (50 mmol/L) and 25 μL of the SPLASH LIPIDOMIX Mass Spec Standard were added. Lipids were then extracted according to Matyash et al. with some modifications.^49^ Details are described in the Supplementary Note 2.

LC-MS/MS measurements were performed on an UltiMate 3000 UHPLC system (Thermo Fisher Scientific, Bremen, Germany) equipped with a reversed-phase ACQUITY UPLC HSS T3 column (1.8 μm, 2.1 × 100 mm, Waters GmbH, Eschborn, Germany) coupled to an orbital trapping mass spectrometer (Q Exactive HF-X, Thermo Fisher Scientific, Bremen, Germany). The UHPLC separation method was adapted from our previous publication,^50^ using water/acetonitrile (4/6; v/v) as mobile phase A and isopropanol/acetonitrile (9/1, v/v) as mobile phase B, both containing 0.1% formic acid and 10 mM ammonium formate. The solvent flow rate was set to 0.26 mL/min, the injection volume was 10 μL, and the column oven temperature was kept constant at 40 °C. Measurements were conducted in the positive- and negative-ion mode. Detailed information about the gradient, ion-source parameters, and mass spectrometric parameters are listed in Tables S1–S3. The raw data are freely available under ftp://massive.ucsd.edu/v08/MSV000095777/.

For data analysis, MS-DIAL software^51^ (version 5.1.230517) was used with the project parameters shown in Table S4. For statistical analysis, Perseus software (version 2.0.10.0) was used,^52^ and for the generation of volcano plots, the online tool VolcaNoseR (https://huygens.science.uva.nl/VolcaNoseR/) was used.^53^ Other graphics were generated with Microsoft Excel. More details can be found in the Supplementary Note 3.

#### Nano-HILIC MS/MS

For nano-HILIC MS/MS, tissue samples were homogenized by bead milling (Mini-Mill PULVERISETTE 23, Fritsch, Idar-Oberstein, Germany). Then, the extraction and purification of GSLs were carried out according to our previous publication.^23^ The SM(d9) 18:1;O2/18:1 species of the SPLASH LIPIDOMIX Mass Spec Standard (Avanti Polar Lipids, Inc.) was used as a reference for relative quantification and was added prior to injection, to account for technical variations.

The samples were analyzed using an UltiMate 3000 RSLCnano system (Thermo Fisher Scientific, Dreieich, Germany) equipped with an Accucore 150 amide-HILIC column (0.075 mm × 150 mm) coupled to an orbital trapping mass spectrometer (Thermo Scientific Q Exactive HF-X). The same method as for our previous publication was used with parameters given in the Supplementary Note 4 and Tables S5 and S6.^23^ The raw data are freely available under ftp://massive.ucsd.edu/v08/MSV000095777/.

To analyze the data, MS-DIAL was employed semiquantitatively for the creation of a GSL database. In the initial step, features were detected by MS-DIAL, applying various parameters for optimal feature detection, using insights gained from a prior data set. The final settings are shown in the Supporting Information in Table S7. Next, we used the MS-DIAL MS/MS search module with neutral losses and ions typical for glycosphingolipids. A list of *m*/*z* values used for this processing step is given in Tables S8 and S9. The features were then inspected manually to create a GSL database. Subsequently, this database was used in MS-DIAL for post identification in positive-ion mode, with parameters outlined in Table S10. More details are described in the Supplementary Note 5.

#### Identification of *F. hepatica* Elongase

*F. hepatica* orthologues of *Homo sapiens* very long-chain fatty acid elongase 1 (NP_001243328.1) were identified by WormBase ParaSite BLAST with standard parameters.^54^ We found four *F. hepatica* elongase genes within the *F. hepatica* proteome in Wormbase ParaSite (PRJNA179522). SMART confirmed the presence of an ELO domain in all of the four proteins.^55^ ELO domains had 33.6–49.0% amino acid identity with the human sequence (aligned in Clustal Omega (v1.2.4).^56^ The spatial distribution of elongase expression (transcripts) in liver fluke tissues was then assessed with help of a liver fluke spatial transcriptomics data set. This data set was generated in a separate study, which is currently under review.^28^ The spatial transcriptomics data set will be available as an online platform upon publication and will allow researchers to search the data set for their needs, as it was done for the elongases in this manuscript.

#### Data Processing

All graphical representations and mass spectra presented were processed using CorelDRAW 2021 software (version 23.1.0.389).

#### Nomenclature

**Figure 8:**
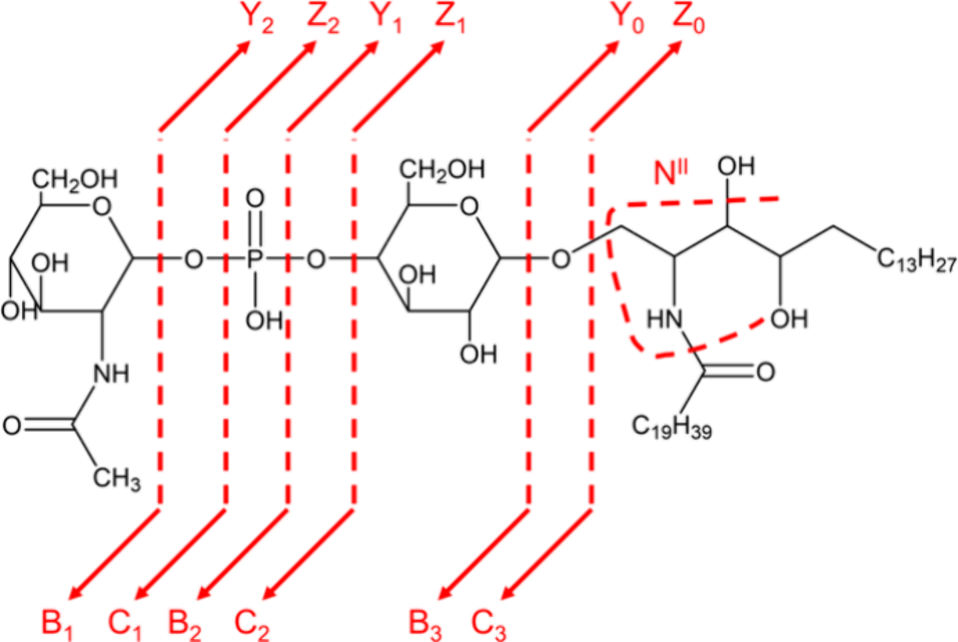
Fragment ion nomenclature after Domon and Costello and Merrill et al. for a glycosphingolipid, consisting of two monosaccharides linked by a phosphate group and a ceramide head group. Cleavage sites are indicated upon HCD fragmentation by dashed lines, resulting in Y-, Z-, B, and C-fragment ions for the glyosidic cleavage of the saccharide units and the N^II^-fragment ion for the sphingoid base. As a sphingoid base, a phytosphingosine is shown exemplarily because glycosphingolipids of parasites often consist of these sphingoid bases.

To describe lipids and GSLs, the shorthand nomenclature of LIPIDMAPS and the nomenclature for glycans are used in this manuscript.^57,58^ Hexoses, like glucose, galactose, or mannose, are abbreviated as Hex. Hexosamines are abbreviated as HexNac and fucose as Fuc. The acidic saccharides *N*-acetylneuraminic acid and *N*-glycolylneuraminic acid are abbreviated as NeuAc and NeuGc, respectively. For example, a GSL with one hexosamine and two hexoses and a ceramide with a d-erythro-hydroxysphinganine and a hydroxypalmitoyl acid is abbreviated as HexNacHex_2_Cer 18:0;O3/h16:0. The fragment ion nomenclature of GSLs after Domon and Costello and Merrill et al. is shown in Figure 8, indicating GSL-specific cleavage sites.^59,60^

## Supporting Information Available

Additional experimental protocols and experimental parameters, data processing steps (Supplementary Notes S1–S6, Tables S1–S10, and Figures S1–S18), identification of GSLs for each sample group (Table S11), and additional results of RP-LC MS/MS experiments (Supplementary Note S8, Figures S19 and S20, and Table S12) (PDF)
In-house created GSL database and RP-LC MS/MS identifications (XLSX)
