# Supplementary material for: Glycolipidomics of Liver Flukes and Host Tissues during Fascioliasis: Insights from Mass Spectrometry Imaging
Source: ACS Infect Dis. 2024 Nov 7;10(12):4233–45. doi: 10.1021/acsinfecdis.4c00551 (PMC11650768; doi:10.1021/acsinfecdis.4c00551)
Supplement: Supplementary file 1 — id4c00551_si_001.pdf [file id4c00551_si_001.pdf]

Supporting Information

Glycolipidomics of liver flukes and host tissues during fascioliasis – insights from mass spectrometry imaging

David Luh,[1] Parviz Ghezellou,[1] Sven Heiles,[2,3] Svenja Gramberg,[4] Simone Haeberlein,[4] Bernhard Spengler[1,*]

[1]Institute of Inorganic and Analytical Chemistry, Justus Liebig University Giessen, 35392 Giessen, Germany

[2]Leibniz-Institut für Analytische Wissenschaften - ISAS - e.V., 44139 Dortmund, Germany

[3]Lipidomics, Faculty of Chemistry, University of Duisburg-Essen, 45141 Essen, Germany

[4]Institute of Parasitology, Biomedical Research Center Seltersberg (BFS), Justus Liebig University Giessen, 35392 Giessen, Germany

* - Corresponding Author, email address: Bernhard.Spengler@anorg.chemie.uni-giessen.de

Supplementary Note 1 – AP-SMALDI MSI

AP-SMALDI measurements were performed with different step sizes down to 5 µm as mentioned for each figure separately. The measurements were performed with a mass range of *m*/*z* 550 – 2200 for rat tissue samples and *m*/*z* 700 -2800 for isolated adult *F*. *hepatica*. The mass resolution was set to 240 000 at *m*/*z* 200. Measurements were performed with 50 laser pulses per pixel at a wavelength of 343 nm. Capillary temperature was set to 250 °C, the S-lens was set to 100 and the acceleration voltage was set to ± 3 kV.

Raw files were recalibrated with ReCal Offline. Ion images were generated with Mirion, the brightness was adjusted for better visualization.[1]

Supplementary Note 2 – Lipid Extraction for HPLC-MS/MS

For lipid extraction, 100 µL ice-cold methanol and 400 µL MTBE were added to each tissue homogenate. The sample was shortly vortexed, followed by shaking for one hour in a Thermomixer C (Eppendorf SE, Hamburg, Germany) at 4 °C and 1000 rpm. Then, 200 µL ice-cold water was added and the sample was centrifuged for 10 min at 1000 g. The upper organic layer was removed and stored in an Eppendorf tube at -20 °C. The extraction procedure was repeated with the remaining aqueous layer by adding a mixture of MTBE/MeOH/water (4/1.2/1, v/v/v). Subsequently, the sample was shaken for one hour at 4 °C and 1000 rpm, followed by centrifugation for 10 min at 1000 g. The organic layers were combined and evaporated under a stream of nitrogen and stored at -80 °C. Samples were resuspended in 500 µL acetonitrile/isopropanol/water (65/30/5, v/v/v). For LC-MS/MS measurements, samples were diluted by using 2 µL of the sample and adding 198 µL of acetonitrile/isopropanol/water (65/30/5, v/v/v).

Supplementary Note 3 – LC-MS/MS data analysis with Perseus software

Identification results obtained by MS-DIAL were normalized to the internal standard and wet-tissue weight, positive- and negative-ion mode data were combined, double annotations were removed while the most intense entry was kept, and then uploaded into Perseus. In Perseus, a log_2-transformation was performed, and missing values were replaced by "0". For revealing significant up- and down-regulation between two biological groups, a two-sided Student's T-tests with a threshold p-value of 0.05 was performed. Volcano plots were generated and the corresponding data matrix was used for the upload to https://huygens.science.uva.nl/VolcaNoseR/. Here, the fold change threshold was set to -1.5 to 1.5 and the significance threshold was set to 1.3.

Supplementary Note 4 – Experimental parameters for nano-HILIC MS/MS analysis

For nano-HILIC MS/MS experiments, solvent A consisted of acetonitrile/methanol (99/1, v/v) and solvent B of methanol/water (8/2, v/v), both with 5 mmol/L ammonium acetate. For injection, the pre-concentration setup was used with microliter-pickup injection. The sample injection volume was 1 µL. Further parameters are described in detail in Table S5/S6.

Supplementary Note 5 – GSL database generation for data dependent acquisition of nano-HILIC MS/MS experiments

For data analysis we conducted MS DIAL feature detection on all samples within this study, identifying 170,583 alignment spots for positive-ion mode and 37,149 for negative-ion mode. Employing MS DIAL feature MS/MS search, tailored explicitly for glycolipid fragments, we identified 9,423 features in positive-ion mode and 1,252 features in negative-ion mode. Through a preliminary manual inspection of the corresponding MS/MS spectra within the MS-DIAL interface, we streamlined the data matrix to 1,154 features in positive- and 1,104 in negative-ion mode. Ultimately, our analysis revealed 212 GSLs

across all samples validated by MS^2 fragmentation patterns in combination with the retention time. 104 GSLs were detected in both polarities, 61 exclusively in positive- and 47 exclusively in negative-ion mode. The comprehensive database, detailed in the Supplementary File 1, encompasses a total of 37 distinct saccharide compositions. Additionally, 57 different compositions are delineated for the ceramide backbone, with a preference for selecting the most intense N^{II}-fragment for ceramide identification. By using MS-DIAL and its available features, it was possible to reduce the time-consuming manual evaluation of data dependent nano-HILIC MS/MS analysis, while simultaneously mitigating researcher dependencies in recognizing GSL fragmentation patterns. Nevertheless, while a fully automated software solution holds the potential to expedite processes further and diminish human errors, our curated database, coupled with additional GSL species, positions MS-DIAL as a robust platform for the comprehensive analysis of nano-HILIC MS/MS data.

Because we aimed for as many identifications as possible for curating the database, multiple annotations for the same feature were obtained. However, precise computations of the signal intensities or areas are a vital requirement for normalization with subsequent relative quantification. Therefore, we reprocessed the nano-HILIC MS/MS data in MS-DIAL with more robust parameters, leading, for example, to less multiple-annotated features. Therefore, our curated database was deployed for post-identification in MS-DIAL. With the more robust parameters, we successfully identified 163 out of 209 GSLs across all samples in the positive-ion mode.

For statistical analysis, areas based on MS-DIAL post-identification in positive-ion mode were normalized to the weight and the reference signal intensity of (SM(d9) 18:1;O2/18:1). For double-identified GSLs, the compound with the lowest mass deviation was kept. Double identification mostly occurred when the singly- and doubly-charged adducts of GSL compounds were both identified or when isobaric species were present. The resulting data matrix was then imported in Perseus. In Perseus, a $\log_2$-transformation was performed, and missing values were replaced by "0". For revealing significant up- and downregulation between two biological groups, a two-sided Student's T-test with a threshold p-value of 0.05 was performed. The volcano plot was generated and the corresponding

data matrix was used for the upload to https://huygens.science.uva.nl/VolcaNoseR/.[2] For the hierarchical clustering, the normalized data was uploaded to https://biit.cs.ut.ee/clustvis/.[3]

The normalized data are presented in a heatmap in Figure S16. It is obvious that the isolated adult *F. hepatica* shows up with various characteristic GSL species, but also for the bile duct of rats in the chronic infection stage, many characteristic GSL species were observed. In Table S11, the different GSLs are listed, with an "x" indicating that this species was identified in the post-identification analysis. The total identification number slightly differs from the previously mentioned 166, because the post-identification was carried out in positive- and additionally in negative-ion mode, whereas for the normalized data only the positive-ion mode was used. For pure post-identification we also applied the peak count filter to 33% and the mass deviation for identification to 0.01 Da and analyzed each biological group separately.

Supplementary Note 6 – Hematoxylin and eosin staining protocol

Hematoxylin and eosin (H&E) staining was performed after AP-SMALDI MSI measurements. First, matrix was rinsed off with ethanol and then the sample was dehydrated for 2 min in ethanol, followed by a 2 min incubation in 70% ethanol. Then, the sample was incubated for 2 min in 40% ethanol followed by a 2 min incubation step in HPLC-grade water. Staining with hematoxylin, washing with tap water for 15 min and staining with 1% Eosin Y solution for 1 min and differentiation with HPLC-grade water for 2 min, 40% ethanol for 2 min, 70% ethanol for 2 min and 100% ethanol for 2 min were subsequently carried out. Clearing with xylol for 2 min and covering with Eukitt and a cover slide completed the staining protocol.

Supplementary Note 7 – Description of H&E-stained tissue sections of rat liver during the acute infection stage

During acute infection with fascioliasis, immature *F. hepatica* (Figure S2 in black) are migrating through the liver parenchyma. These parasites cause mechanical damage as well as damage caused by shedding digestive enzymes, resulting in visible migratory tracts. The centers of these tracts are marked in orange in Figure S2 and are rich in leukocyte infiltrates. The centers are surrounded by necrotic liver tissue, which is highlighted in Figure S2 in blue. This necrotic tissue is often still rich in leukocyte infiltrates. Additionally, several blood vessels are highlighted in red, with perivascular leukocyte infiltrates, marked in purple as shown in Figure S2. Furthermore, several areas are highlighted in white. These areas may represent swollen liver tissue.

Supplementary Note 8 – Supporting RP-LC MS/MS data

We investigated alterations in the hepatic lipidome during acute *F. hepatica* infection compared to healthy controls. As depicted in Figure S19, the total number of identified lipid species exhibits a remarkable similarity with subtle distinctions. As an example, in the acutely infected sample, eleven LPE-O species were identified, whereas in the control sample only six were identified. However, statistical analysis revealed more profound differences. A significant downregulation ($p < 0.05$) was observed in glycerophospholipids such as phosphatidylcholines (PCs), phosphatidylserines (PSs), and phosphatidylinositols (PIs), along with their corresponding ether-lipid classes (Figure S7). Interestingly, exceptions to this trend were noted, highlighting an upregulation of phosphatidylethanolamines (PEs), ether-linked phosphatidylethanolamines (PE-Os), and lysophosphatidylethanolamines (LPE-Os). Furthermore, we identified a significant upregulation in glycerolipids, encompassing triacylglycerols (TGs), diacylglycerols (DGs), as well as free fatty acids (FAs), oxidized free fatty acids (FA-Os), and ceramides (Cer) in the samples from acutely infected subjects. In contrast, cardiolipins (CLs) and sphingomyelins (SMs) exhibited a distinct downregulation. The summarized results are visually

represented in Figure S7, while the corresponding volcano plot in Figure S20 illustrates the down- and upregulated lipid species in acutely infected samples compared to the control. Highlighted in green are the two lipids PC 36:4 and TG 58:8, which are shown in the main manuscript in the RG-overlay image (Figure 4b). Furthermore, the volcano plot also includes 363 unknown lipid species. These species are annotated from the RIKEN database, lacking specificity regarding molecular species.

With respect to the rat liver of the chronic infection stage, only 11 lipid species appeared significantly up- or downregulated ($p < 0.05$) compared to the same-aged healthy control, with the species listed in Table S12. Therefore, we assume that a normalization in the lipid metabolism is almost reached at the chronic time point of infection, and that we rather observe biological variations and measurement inaccuracies. Interestingly, a statistical comparison between the bile duct at the chronic infection stage and the rat liver of the healthy control (20-21 weeks of age) revealed, among others, eleven significantly ($p < 0.05$) upregulated LPE-O species. Together with the outcome of non-significant changes in LPE-O species between the bile duct sample and the rat liver at the acute infection stage, this molecular class can potentially be linked to the host-parasite interaction. Our supplementary file 1 encompasses a comprehensive lipid database, derived from our RP-LC MS/MS measurements, providing reference data.

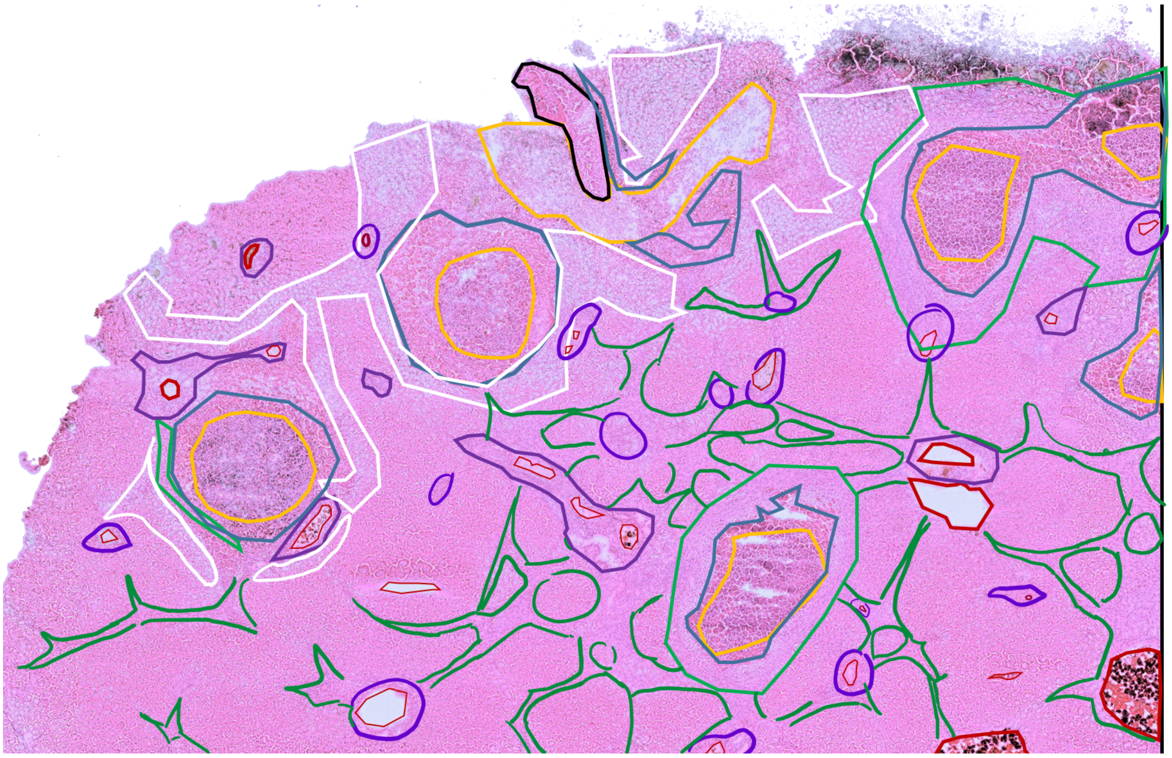

Figure S1: H&E-stained tissue section of a rat liver during the acute infection stage. Different histological regions are highlighted. The migrating immature *F. hepatica* in black, connective tissue in green, foci of migratory tracts in orange, rim of migratory tracts in blue, blood vessels in red, perivascular tissue in purple and swollen hepatocytes in white.

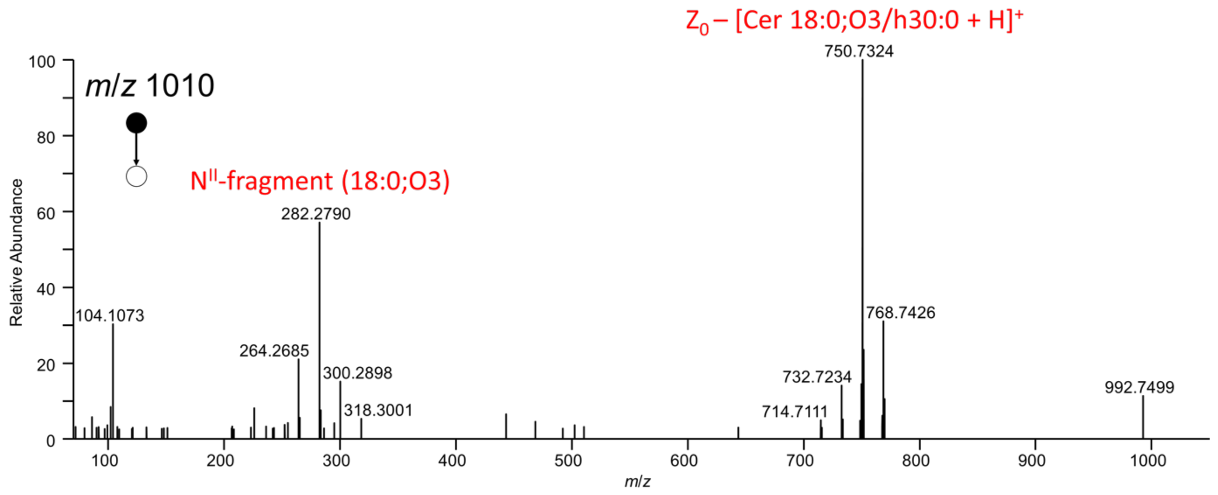

Figure S2: Tandem mass spectrum for HPO_3-Hex-Cer 18:0;O3/h30:0 ($[M+H]^+$ at *m/z* 1010.76). The fragment ion at *m/z* 282.28 corresponds to the N^{II}-fragment. The fragment ion at *m/z* 750.73 represents the Z_0-fragment ion with a neutral loss of 260.03 Da (Hex+HPO_3) from the parent ion.

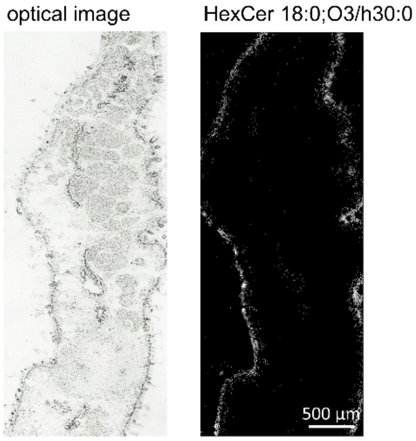

Figure S3: (left) Optical image of a transversal *F. hepatica* tissue section. (right) Single-ion image showing the distribution of HexCer 18:0;O3/h30:0 ([M+K]+ at *m/z* 968.7527) across the tegument.

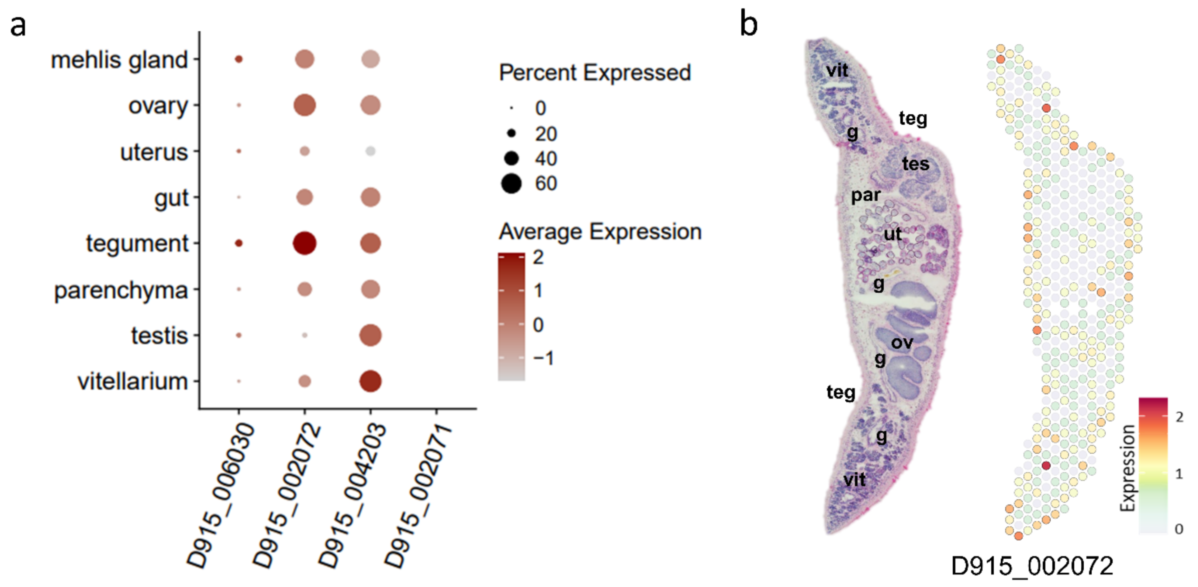

Figure S4: Spatial transcriptomics data for the elongase genes D915_006030, D915_002072, D915_004203 and D915_002071 in adult *F. hepatica*. (a) DotPlot showing elongase expression profiles across eight liver fluke tissues. Dot color encodes the average expression level in a given tissue. Dot size encodes the percentage of spots assigned to a tissue that have captured elongase transcripts. (b) H&E-stained tissue section and corresponding spatial projection showing the expression pattern of D915_002072. Expression level encoded by color (grey = low, red = high). g: gut, ov: ovary, par: parenchyma, teg: tegument, tes: testis, ut: uterus, vit: vitellarium.

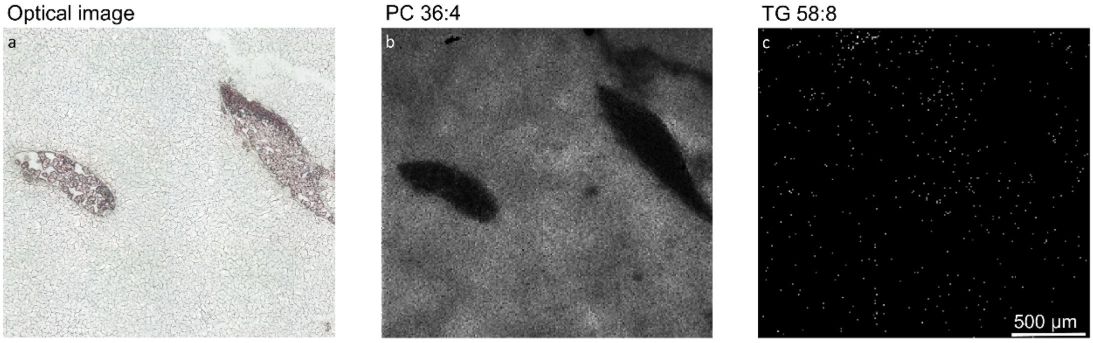

Figure S5: (a) Optical image of a control liver tissue section from a healthy rat, which was the same age as the rat of the acute infection stage. (b) Single-ion image showing PC 36:4 ($[M+K]^+$ at *m/z* 820.5253). (c) Single-ion image of TG 58:8 ($[M+K]^+$ at *m/z* 969.7308). The AP-SMALDI MSI measurement was performed with 10 µm step size.

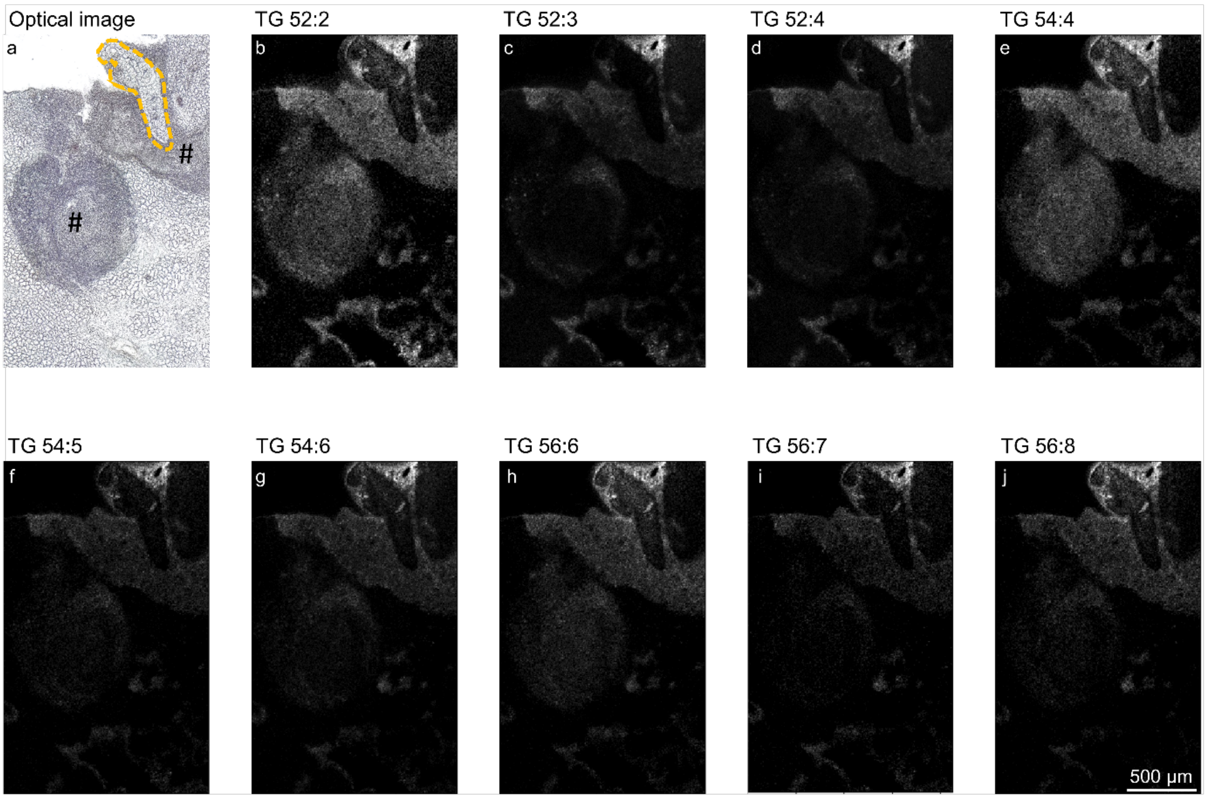

Figure S6: (a) Optical image of liver tissue section from a rat at the acute infection stage. The migrating *F. hepatica* is outlined by an orange-dotted line, and tissue lesions are marked by a hash. (b) Single-ion image showing TG 52:2 ($[M+K]^+$ at *m/z* 897.7308). (c) Single-ion image showing TG 52:3 ($[M+K]^+$ at *m/z* 895.7151). (d) Single-ion image showing TG 52:4 ($[M+K]^+$ at *m/z* 893.6995). (e) Single-ion image showing TG 54:4 ($[M+K]^+$ at *m/z* 921.7308). (f) Single-ion image showing TG 54:5 ($[M+K]^+$ at *m/z* 919.7308). (g) Single-ion image showing TG 54:6 ($[M+K]^+$ at *m/z* 919.7151). (h) Single-ion image showing TG 56:6 ($[M+K]^+$ at *m/z* 945.7308). (i) Single-ion image showing TG 56:7 ($[M+K]^+$ at *m/z* 943.7151). (j) Single-ion image showing TG 56:8 ($[M+K]^+$ at *m/z* 941.6995). The AP-SMALDI MSI measurement was performed with 7 µm step size.

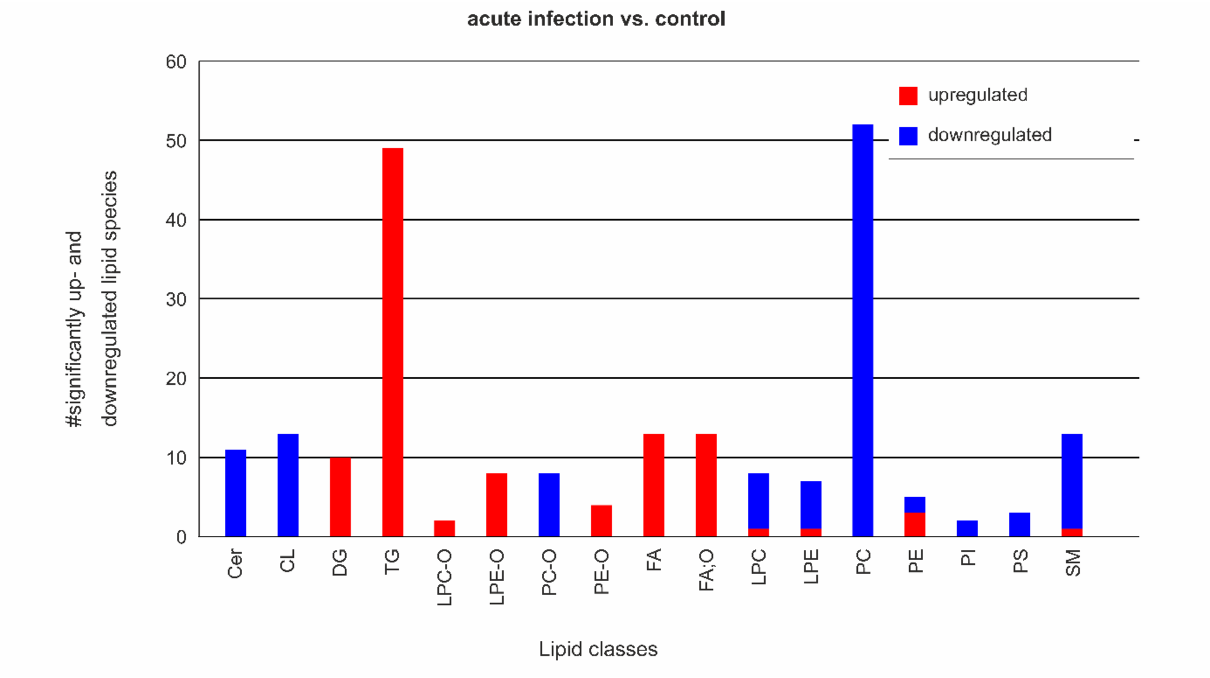

Figure S7: Overview of the number of significantly ($p < 0.05$) up- or downregulated lipid classes in rat liver during the acute infection stage compared to healthy controls. Data is based on semi-quantitative RP-LC MS/MS data analysis. A two-sided Student's T-tests with a threshold p-value of 0.05 was performed. More details are described in the supplementary note 3.

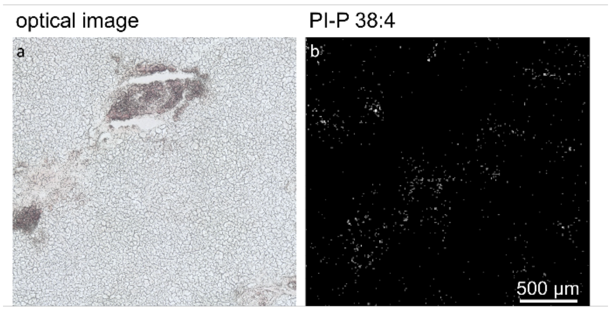

Figure S8: (a) Optical image of a control liver tissue section from a healthy rat, which was the same age as the rat of the acute infection stage. (b) Single-ion image showing PI-P 38:4 ([M-H]$^-$ at *m/z* 869.5544. The AP-SMALDI MSI measurement was performed with 10 µm step size.

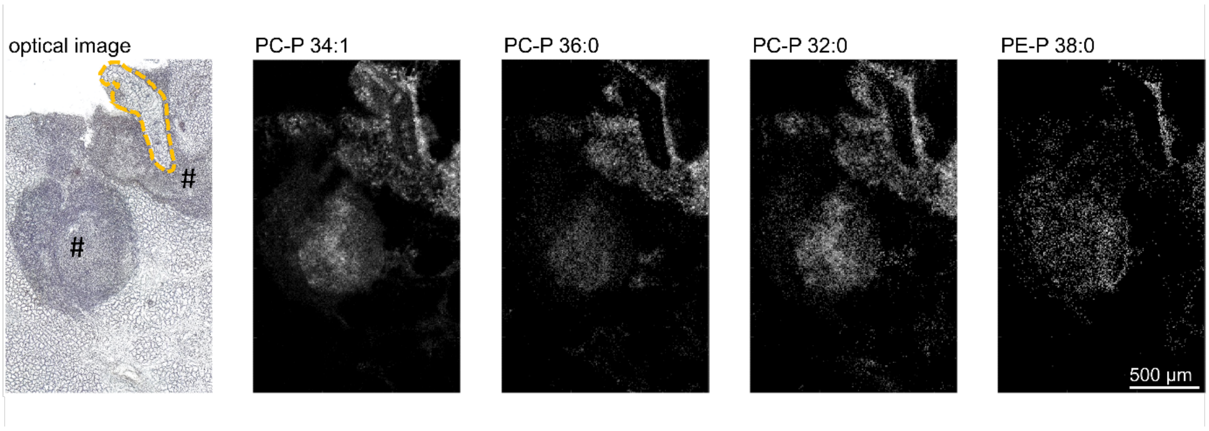

Figure S9: (a) Single-ion images showing PC-P 34:1 ([M+K]$^+$ at *m/z* 782.5460). The migrating *F. hepatica* is outlined by an orange-dotted line, and tissue lesions are marked by a hash. (b) Single-ion image showing PC-P 34:1 ([M+K]$^+$ at *m/z* 782.5460). (c) Single-ion image showing PC-P 36:0 ([M+K]$^+$ at *m/z* 812.5930). (d) Single-ion image showing PC-P 32:0 ([M+K]$^+$ at *m/z* 756.5304). (e) Single-ion image showing PE-P 38:0 ([M+Na]$^+$ at *m/z* 782.6034). The AP-SMALDI MSI measurement was performed with 7 µm step size.

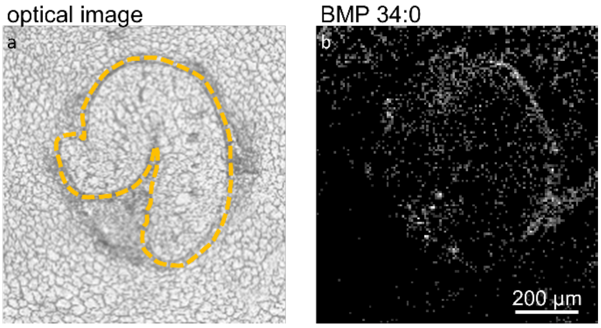

Figure S10*:* (a) Optical image of liver tissue section from a rat at the acute infection stage. The migrating *F*. *hepatica* is outlined by an orange dotted line. (b) Single-ion image showing BMP 34:0 ([M-H]$^-$ at *m/z* 749.5325). The AP-SMALDI MSI measurement was performed with 7 µm step size.

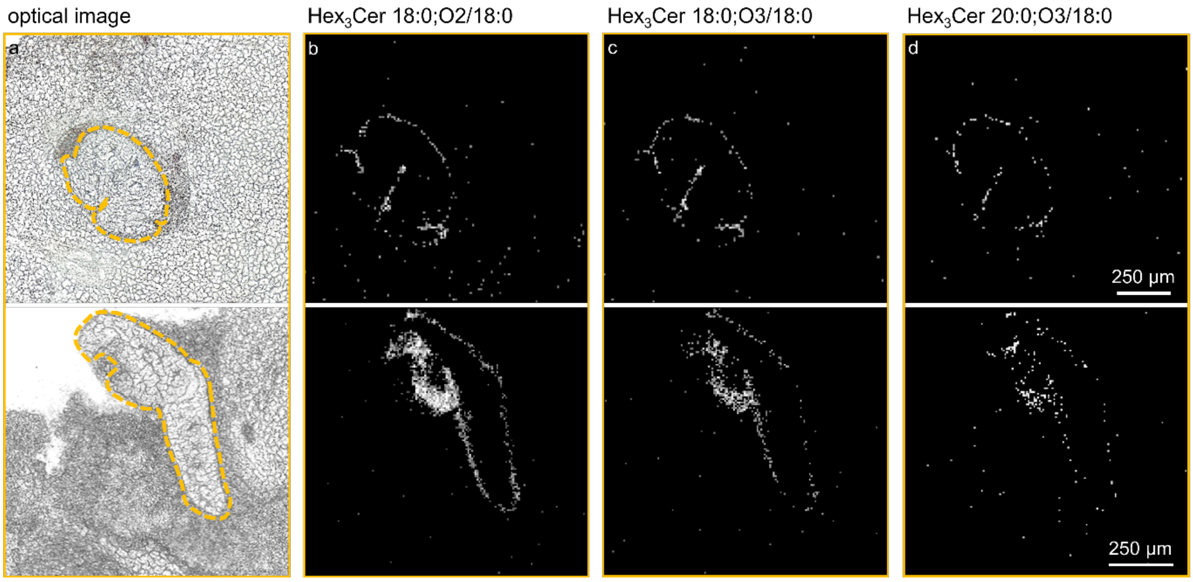

Figure S11: (a) Optical image of 1. replicate in the top row and 2. replicate in the bottom row. The migrating *F. hepatica* is outlined by an orange-dotted line. (b) Single-ion images for Hex_3Cer 18:0;O2/18:0 ([M+K]$^+$ at *m/z* 1092.6787). (c) Single-ion images for Hex_3Cer 18:0;O3/18:0 ([M+K]$^+$ at *m/z* 1108.6738). (d) Single-ion images for Hex_3Cer 20:0;O3/18:0 ([M+K]$^+$ at *m/z* 1136.7057) for each migrating *F. hepatica*. The AP-SMALDI MSI measurement in the upper row was performed with 10 µm step size. The AP-SMALDI MSI measurement in the bottom row was performed with 7 µm step size.

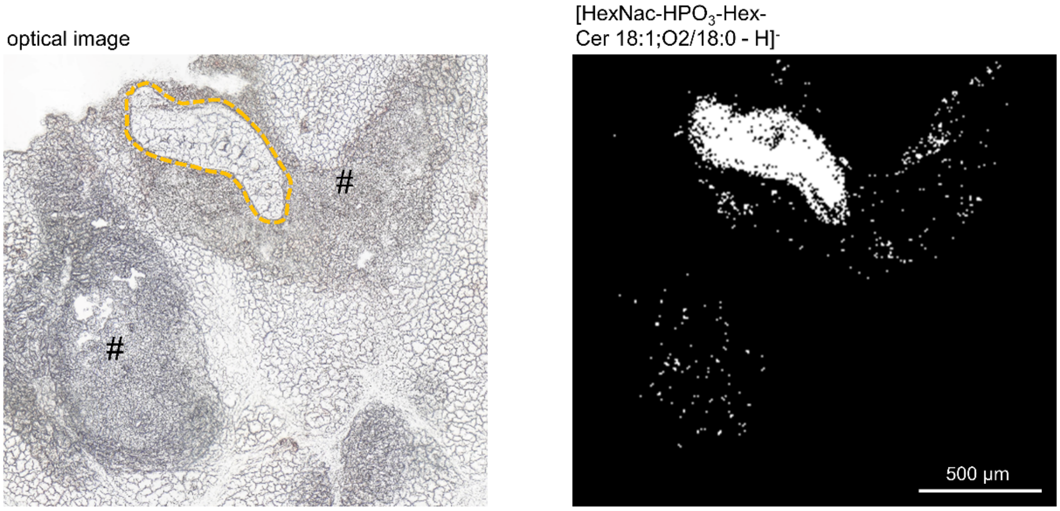

Figure S12: (a) Optical image of liver tissue section from a rat at the acute infection stage. The migrating *F. hepatica* is outlined by an orange dotted line. (b) Single-ion image showing HexNac-HPO_3-Hex-Cer 18:1;O2/18:0 ($[M-H]^-$ at *m/z* 1027.6452). The ion image is maximally overexposed to enhance the visibility of the low-intensity signals distributed across the tissue lesions. The AP-SMALDI MSI measurement was performed with 7 µm step size.

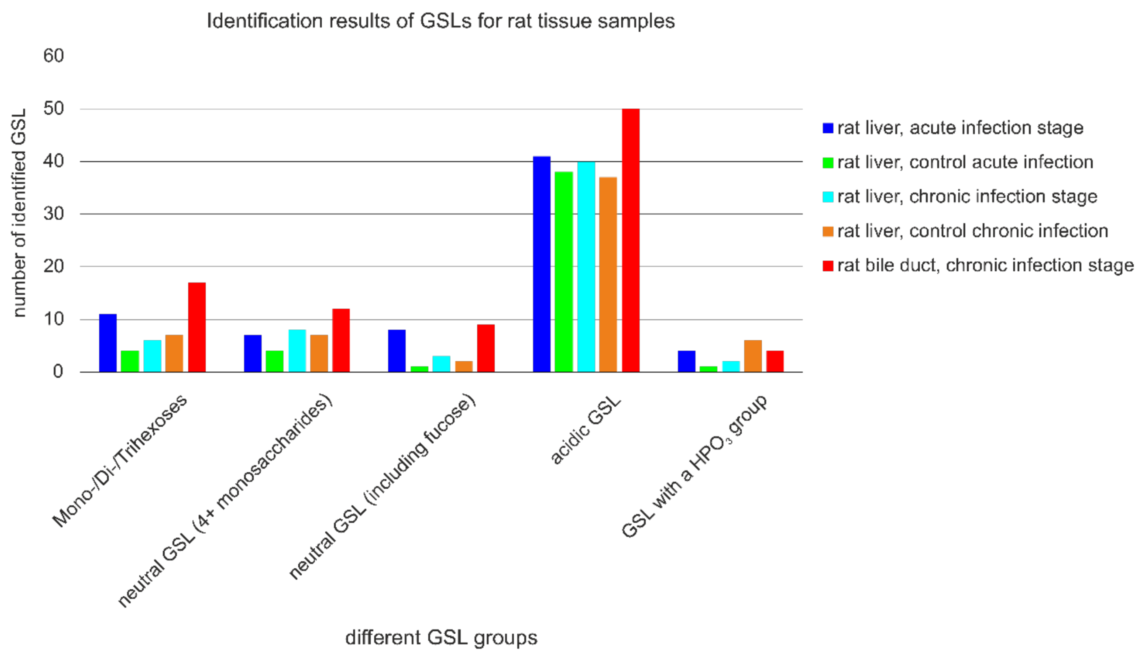

Figure S13: Total number of GLS species in host tissue samples identified through nano-HILIC MS/MS analysis with the data analysis described in Supplementary note 5. The identification results were grouped, namely as mono-/di-/trihexoses, neutral GSLs with more than four saccharide units, neutral GSLs containing at least one fucose moiety, acidic GSLs and GSLs bearing with a phosphate group.

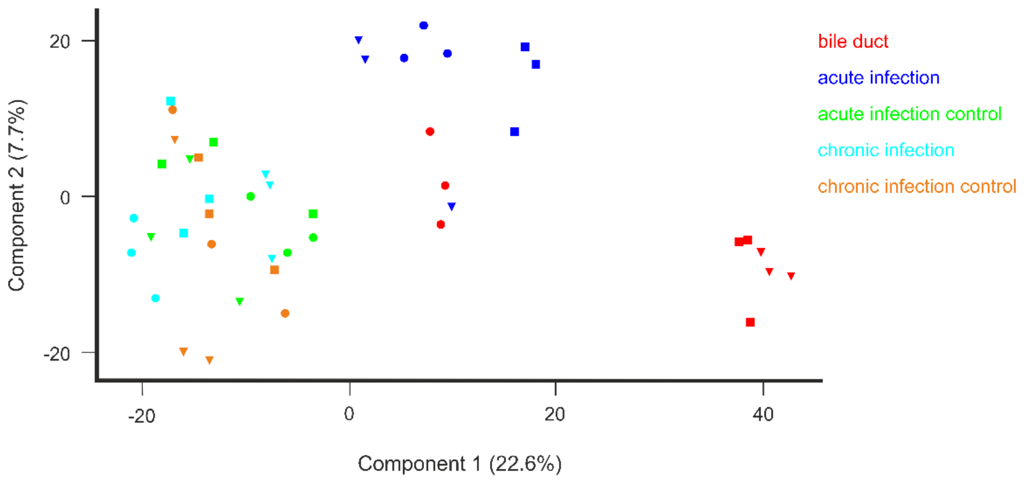

Figure S14: PCA Plot created with normalized nano-HILIC post-identification results for GSL species in positive-ion mode. Color-coded for biological groups with the bile duct samples in red, the rat liver of acute infection stage in blue, the corresponding control in green, the rat liver of chronic infection stage in turquoise and the corresponding control in yellow. Different symbols within one biological group represent biological replicates. Consequently, the same symbol within one biological group represents a technical replicate.

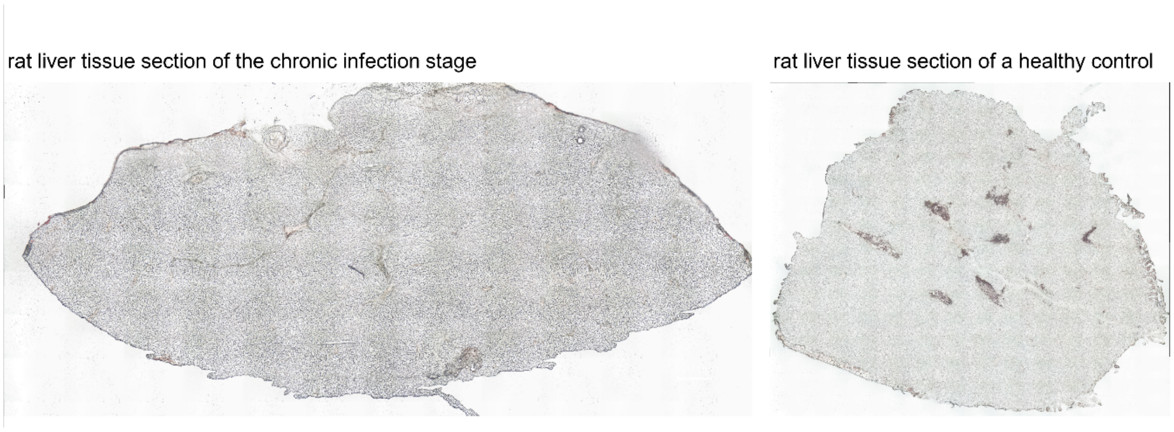

Figure S15: Optical images of rat liver tissue sections. (Left) Rat liver tissue section of the chronic *F. hepatica* infection stage (left). (Right) rat liver tissue section of a healthy control. Several blood vessels can be seen as dark areas in this tissue section.

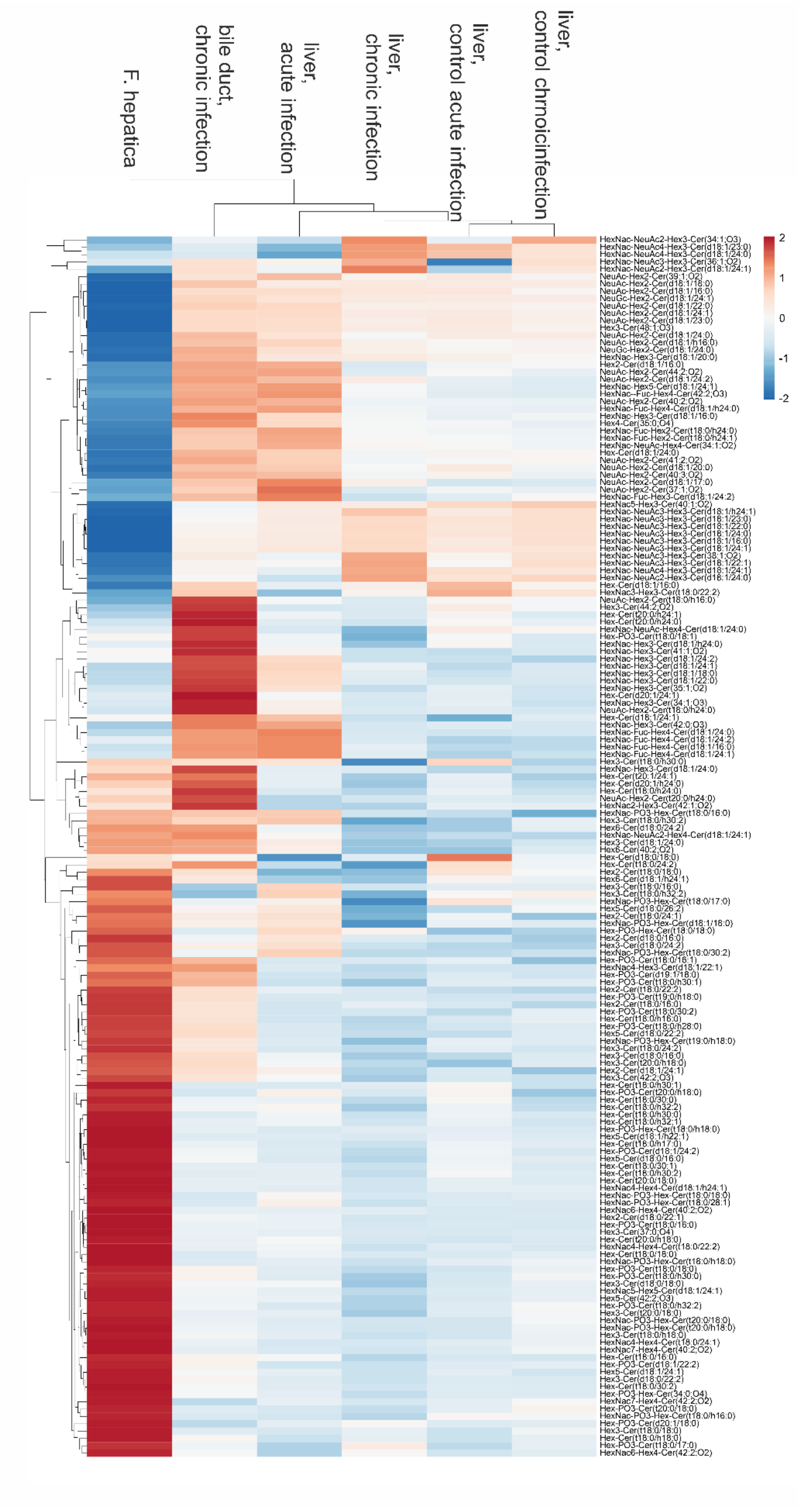

Figure S16: Hierarchical Clustering of normalized GSL profiling data. Red color represents enhanced signals, and blue color reduced signals within the different groups, respectively. The pre-processing

options “row centering” and “unit variance row scaling” were chosen. The heatmap was then generated with following parameters: Clustering distance for rows and columns: Elucidan. Clustering method for rows and columns: average. Tree ordering for rows: tightest cluster first.

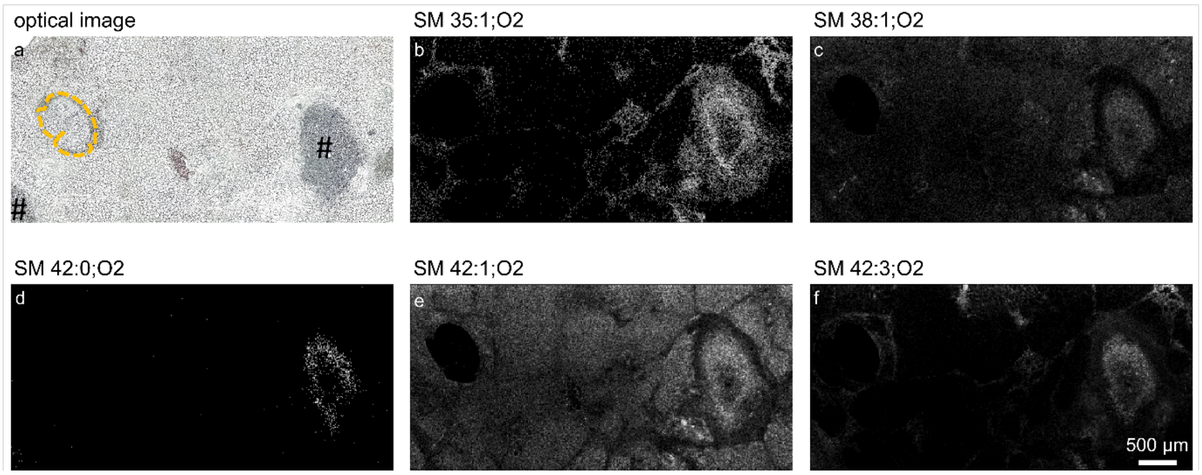

Figure S17: (a) Optical image of a liver tissue section from rat at the acute infection stage. The migrating *F. hepatica* is outlined by orange-dotted line, and tissue lesions are marked by a hash. (b) Single-ion image showing SM 35:1;O2 ($[M+Na]^+$ at *m/z* 739.5725). (c) Single-ion image showing SM 38:1;O2 ($[M+Na]^+$ at *m/z* 809.6507). (d) Single-ion image showing SM 42:0;O2 ($[M+Na]^+$ at *m/z* 839.6976). (e) Single-ion image showing SM 42:1;O2 ($[M+Na]^+$ at *m/z* 837.6820). (f) Single-ion image to (a) showing SM 42:3;O2 ($[M+Na]^+$ at *m/z* 8333.6507). The AP-SMALDI MSI measurement was performed with 10 µm step size.

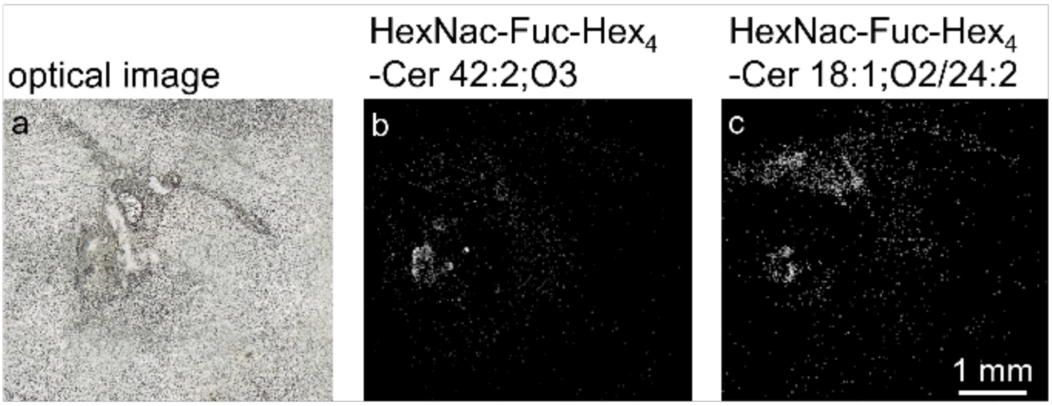

Figure S18: (a) bile duct tissue section from rat at the chronic infection stage. (b) Single-ion image showing HexNacFucHex$_4$Cer 42:2;O3 ($[M+K]^+$ at *m/z* 1699.9388). (c) Single-ion image showing HexNacFucHex$_4$Cer 18:1;O2/24:2 ($[M+K]^+$ at *m/z* 1681.9191. The AP-SMALDI MSI measurement was performed with 20 µm step size in continuous mode.

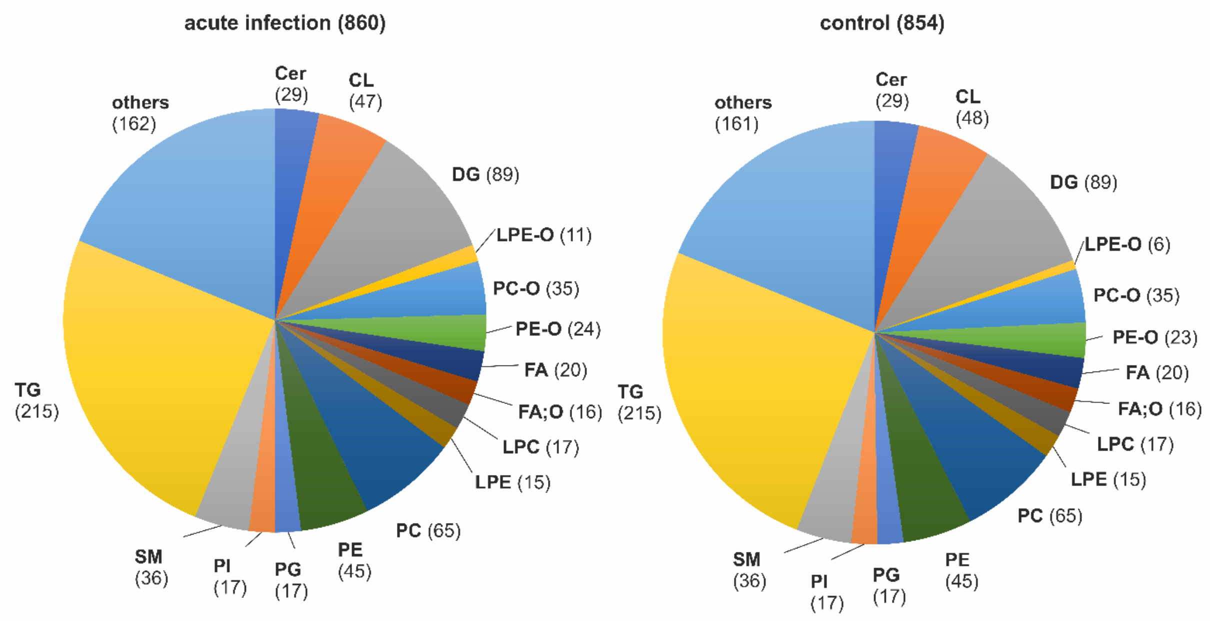

Figure S19: Pie charts showing the identified lipid species using RP-LC MS/MS analysis of liver of rat at the acute infection stage (left)) and liver of healthy controls at the same age (right). “Others” include acylsphingomyelin, bile acids, bismonoacylglycerophosphate, acylcarnitine, sterols, prenol lipids, (di)lysocardiolipin, oxidized triglyceride, GM3, N-acyl-lysophosphatidylethanolamine, lysophosphatidic acid, lysophosphatidylglycerol, lysophosphatidylinositol, lysophosphatidylserine, monoacylglycerol, fatty amides, oxidized phosphatidylethanolamine, oxidized phosphatidylinositol, oxidized triglyceride, phosphatidic acid, phosphatidylethanol, phosphatidylmethanol, phosphatidylserine, sulfatide, sulfonolipid sterol sulfate.

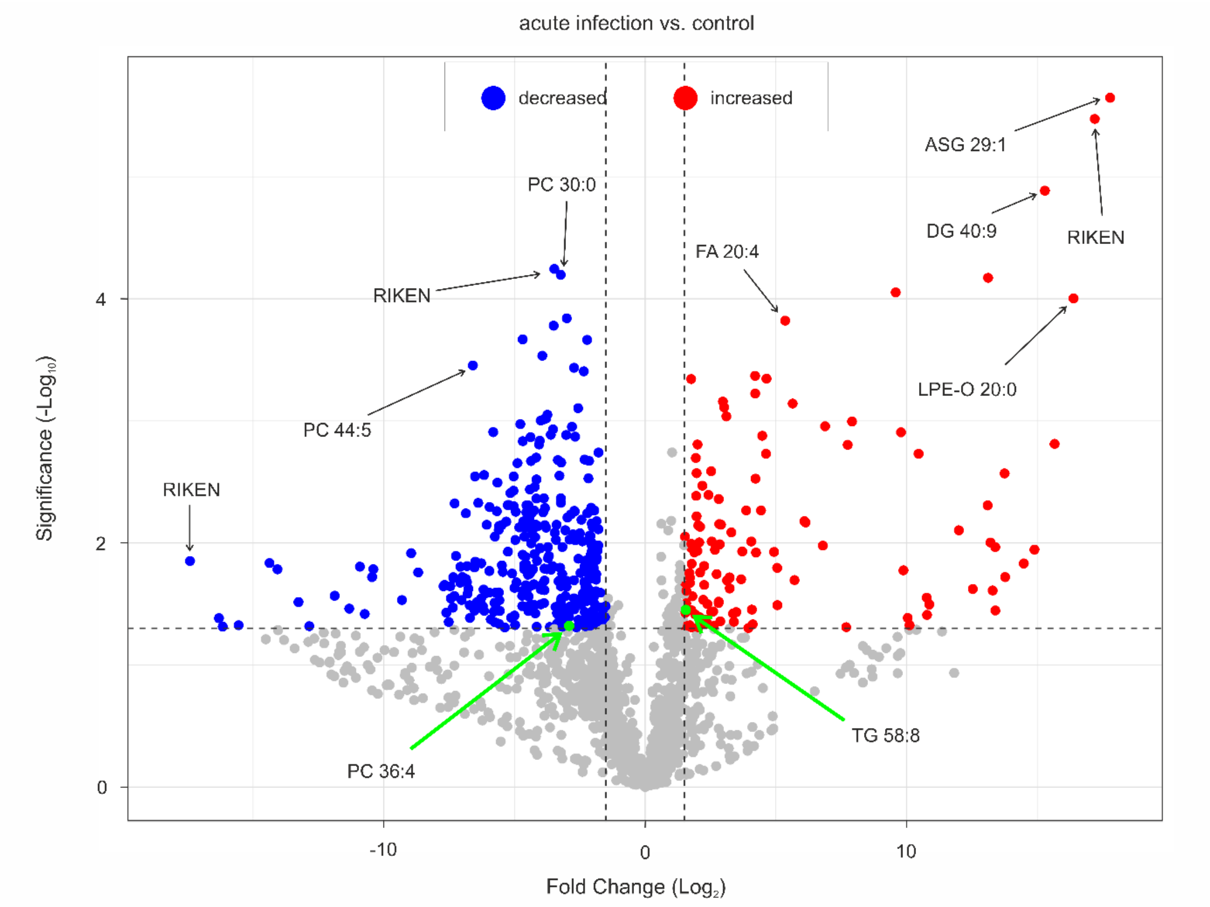

Figure S20: Volcano plot including all data points from RP-LC MS/MS analysis after MS-DIAL analysis and normalization, revealing pronounced differences between the acute infection stage and the corresponding control. The fold-change threshold was set to -1.5 to 1.5 and the significance threshold was set to $p<0.05$.

Table S1: Gradient for RP-LC MS/MS analysis.

Time / min	Mobile Phase A / %	Mobile Phase B / %
**0**	68	32
**1.5**	68	32
**4**	55	45
**5**	48	52
**8**	42	58
**11**	34	66
**14**	30	70
**18**	25	75
**21**	3	97
**25**	3	97
**26**	68	32
**32**	68	32

Table S2: Heated electrospray ionization (HESI-II) source parameters for RP-LC MS/MS analysis.

Parameter	Setting
**Spray voltage / kV**	±3.5
**Capillary temperature / °C**	300 (+), 320 (-)
**Sheath gas / a.u**	40 (+), 45 (-)
**Aux gas / a.u.**	12
**Sweep gas / a.u.**	2
**S-Lens**	55
**Auxiliary heater temperature / °C**	300

Table S3: Full scan (data-dependent MS/MS) acquisition method parameters for RP-LC MS/MS analysis.

Parameter	Setting
**Resolution**	120000 (45000)
**Automatic Gain Control**	$1\cdot10^6$ ($2\cdot10^5$)
**Max. injection time / ms**	250 (75)
**Top N**	15
**Scan range**	300 – 1800 (automatic)
**Isolation window / m/z**	1.0
**NCE**	25; 30
**Intensity threshold**	$1.3\cdot10^4$
**Dynamic exclusion / s**	8.0

Table S4: MS-DIAL settings for the data analysis of reversed phase RP-LC MS/MS experiments.

Task	Setting	Value
**Data collection**	MS 1 tolerance	0.01 Da
	MS2 tolerance	0.025 Da
	Retention time begin	0 min
	Retention time range end	25 min
	MS1 mass range begin	200 Da
	MS1 mass rage end	1800 Da
	MS/MS mass range begin	0 Da
	MS/MS mass range end	2000 Da
**Peak detection**	Minimum peak height	1000 amplitude
	Mass slice width	0.4
	Smoothing method	Linear weighted moving average
	Smoothing level	8 scans
	Minimum peak width	5 scans
**Spectrum deconvolution**	Sigma window value	0.5
	MS/MS abundance cut off	0 amplitude
	Exclude after precursor ion	check
	Keep the isotopic ions until	1
**Identification (SplashLipidomix)**	Accurate mass tolerance (MS1)	0.01 Da
	Retention time tolerance	4 min
	Use retention time for scoring	Check
	Use retention time for filtering	Check
**Identification (Lipid Database)**	Accurate Mass tolerance (MS1)	0.01 Da
	Accurate mass tolerance (MS2)	0.025 Da
	Retention time tolerance	100 min
**Alignment parameters**	Reference file	QC_pos_5 / QC_neg_5
	Retention time tolerance	2 min
	MS1 tolerance	0.01 Da
	Retention time factor	0.5
	MS1 factor	0.5
	Peak count filter	0%
	N% detected in at least one group	80%
	Remove features based on blank information	Check
	Sample max / blank average	5 fold change

Table S5: nano-HILIC Parameter used for separation of glycosphingolipids.

**Property**	**Setting**
Mobile Phase A	acetonitrile/methanol (99/1, v/v), 5 mM AmAc
Mobile Phase B	methanol/water (8/2, v/v), 5 mM AmAc
Loading Solvents	same as starting conditions
Injection volume	1 µL
Loading Time	0.5 minutes
Gradient	5% B for 5 minutes, to 95% B in 45 minutes, hold 95% B for 10 minutes, to 5% B in 5 minutes, hold 5% B for 35 minutes
Oven temperature	40 °C
Autosampler temperature	4 °C
Loading flow rate	30 µL/min
NC flow rate	300 nL/min

Table S6: MS and MS/MS parameters (in brackets) used for identification of GSL after nano-HILIC separation.

**Parameter**	**Setting**
Spray voltage / kV	±2.0
Capillary temperature/ °C	200
S-lens setting	100
Mass resolution at *m/z* 200	120000 (45000)
MS AGC target	$3 \cdot 10^6$ ($1 \cdot 10^5$)
MS max. injection time/ ms	100 (100)
Scan range	*m/z* 500 - 2000
Top N	15
Isolation window / Δ *m/z*	2.0
Stepped NCE	20; 25; 30
Dynamic exclusion / s	6.0

Table S7: MS-DIAL settings for the initial data analysis of the nano-HILIC MS/MS experiments. Parameters were chosen such that many features were detected. Several features were detected multiple times.

Task	Setting	Value
**Data collection**	MS 1 tolerance	0.05 Da
	MS2 tolerance	0.1 Da
	Retention time begin	0 min
	Retention time range end	60 min
	MS1 mass range begin	500 Da
	MS1 mass rage end	2000 Da
	MS/MS mass range begin	0
	MS/MS mass range end	2000 Da
**Peak detection**	Minimum peak height	5000 amplitude
	Mass slice width	0.005
	Smoothing method	Linear weighted moving average
	Smoothing level	2 scans
	Minimum peak width	3 scans
**Spectrum deconvolution**	Sigma window value	0.1
	MS/MS abundance cut off	0 amplitude
	Exclude after precursor ion	Check
	Keep the isotopic ions until	3
**Identification (SplashLipidomix)**	Accurate mass tolerance (MS1)	0.01
	Retention time tolerance	100
**Alignment parameters**	Reference file	QC_pos_5 / QC_neg_5
	Retention time tolerance	2 min
	MS1 tolerance	0.01 Da
	Retention time factor	0.5
	MS1 factor	0.5
	Peak count filter	0%
	N% detected in at least one group	0%

Table S8: MS/MS search parameters in MS-DIAL for the positive-ion mode. Neutral losses or product ions are listed, including the common saccharides for GSL and the mass values for the typical N[II]-fragments.

Species	mass / u	Tolerance / Da	abundance	type
**Fuc**	146.057908	0.005	0.01	NeutralLoss
**Fuc**	147.065184	0.005	0.01	ProductIon
**Hex**	162.052823	0.005	0.01	NeutralLoss
**Hex**	163.060099	0.005	0.01	ProductIon
**Fuc**	164.068473	0.005	0.01	NeutralLoss
**Fuc**	165.075749	0.005	0.01	ProductIon
**Hex**	180.063388	0.005	0.01	NeutralLoss
**Hex**	181.070664	0.005	0.01	ProductIon
**HexNac**	203.079372	0.005	0.01	NeutralLoss
**HexNac**	204.086648	0.005	0.01	ProductIon
**HexNac**	221.089937	0.005	0.01	NeutralLoss
**HexNac**	222.097213	0.005	0.01	ProductIon
**16:2;O2**	234.221619	0.005	0.01	ProductIon
**16:1;O2**	236.237269	0.005	0.01	ProductIon
**16:0;O2**	238.252919	0.005	0.01	ProductIon
**Hex + SO_3**	242.009636	0.005	0.01	NeutralLoss
**17:2;O2**	248.237269	0.005	0.01	ProductIon
**17:1;O2**	250.252919	0.005	0.01	ProductIon
**16:1;O3**	252.232184	0.005	0.01	ProductIon
**17:0;O2**	252.268569	0.005	0.01	ProductIon
**16:0;O3**	254.247834	0.005	0.01	ProductIon
**16:0;O2+H_2O**	256.263484	0.005	0.01	ProductIon
**Hex + SO3**	260.020201	0.005	0.01	NeutralLoss
**18:2;O2**	262.252919	0.005	0.01	ProductIon
**18:1;O2**	264.268569	0.005	0.01	ProductIon
**17:1;O3**	266.247834	0.005	0.01	ProductIon
**18:0;O2**	266.284219	0.005	0.01	ProductIon
**17:0;O3**	268.263484	0.005	0.01	ProductIon
**16:1;O3+H_2O**	270.242749	0.005	0.01	ProductIon
**17:0;O2+H_2O**	270.279134	0.005	0.01	ProductIon
**16:0;O3+H_2O**	272.258399	0.005	0.01	ProductIon
**16:0;O2+2 H_2O**	274.274049	0.005	0.01	ProductIon
**19:2;O2**	276.268569	0.005	0.01	ProductIon
**19:1;O2**	278.284219	0.005	0.01	ProductIon
**18:1;O3**	280.263484	0.005	0.01	ProductIon
**19:0;O2**	280.299869	0.005	0.01	ProductIon
**18:0;O3**	282.279134	0.005	0.01	ProductIon
**HexNac + SO_3**	283.036185	0.005	0.01	NeutralLoss

**17:1;O3+H_2O**	284.258399	0.005	0.01	ProductIon
**18:0;O2+H_2O**	284.294784	0.005	0.01	ProductIon
**17:0;O3+H_2O**	286.274049	0.005	0.01	ProductIon
**16:1;O3+2 H_2O**	288.253314	0.005	0.01	ProductIon
**17:0;O2+2 H_2O**	288.289699	0.005	0.01	ProductIon
**16:0;O3+2 H_2O**	290.268964	0.005	0.01	ProductIon
**20:2;O2**	290.284219	0.005	0.01	ProductIon
**NeuAc**	291.095415	0.005	0.01	NeutralLoss
**NeuAc**	292.102692	0.005	0.01	ProductIon
**20:1;O2**	292.299869	0.005	0.01	ProductIon
**t19:1**	294.279134	0.005	0.01	ProductIon
**20:0;O2**	294.315519	0.005	0.01	ProductIon
**t19:0**	296.294784	0.005	0.01	ProductIon
**18:1;O3+H_2O**	298.274049	0.005	0.01	ProductIon
**19:0;O2+H_2O**	298.310434	0.005	0.01	ProductIon
**18:0;O3+H_2O**	300.289699	0.005	0.01	ProductIon
**HexNac + SO_3**	301.04675	0.005	0.01	NeutralLoss
**17:1;O3+2 H_2O**	302.268964	0.005	0.01	ProductIon
**18:0;O2+2 H_2O**	302.305349	0.005	0.01	ProductIon
**17:0;O3+2 H_2O**	304.284614	0.005	0.01	ProductIon
**NeuGc**	307.09033	0.005	0.01	NeutralLoss
**NeuGc**	308.097606	0.005	0.01	ProductIon
**20:1;O3**	308.294784	0.005	0.01	ProductIon
**NeuAc**	309.10598	0.005	0.01	NeutralLoss
**NeuAc**	310.113257	0.005	0.01	ProductIon
**20:0;O3**	310.310434	0.005	0.01	ProductIon
**19:2;O2+2 H_2O**	312.289699	0.005	0.01	ProductIon
**20:0;O2+H_2O**	312.326084	0.005	0.01	ProductIon
**19:0;O3+H_2O**	314.305349	0.005	0.01	ProductIon
**18:1;O3+2 H_2O**	316.284614	0.005	0.01	ProductIon
**19:0;O2+2 H_2O**	316.320999	0.005	0.01	ProductIon
**18:0;O3+2 H2O**	318.300264	0.005	0.01	ProductIon
**22:2;O2**	318.315519	0.005	0.01	ProductIon
**22:1;O2**	320.331169	0.005	0.01	ProductIon
**22:0;O2**	322.346819	0.005	0.01	ProductIon
**NeuGc**	325.100895	0.005	0.01	NeutralLoss
**2 x Hex**	325.112922	0.005	0.01	ProductIon
**NeuGc**	326.108171	0.005	0.01	ProductIon
**20:1;O3+H_2O**	326.305349	0.005	0.01	ProductIon
**20:1;O2+2 H_2O**	328.320999	0.005	0.01	ProductIon
**19:1;O3+2 H_2O**	330.300264	0.005	0.01	ProductIon
**20:0;O2+2 H_2O**	330.336649	0.005	0.01	ProductIon
**19:0;O3+2 H_2O**	332.315914	0.005	0.01	ProductIon
**22:1;O3**	336.326084	0.005	0.01	ProductIon
**22:1;O2+H_2O**	338.341734	0.005	0.01	ProductIon

**22:0;O2+H_2O**	340.357384	0.005	0.01	ProductIon
**2 x Hex**	342.11621	0.005	0.01	NeutralLoss
**20:1;O3+2 H_2O**	344.315914	0.005	0.01	ProductIon
**20:0;O3+2 H_2O**	346.331564	0.005	0.01	ProductIon
**22:1;O3+H_2O**	354.336649	0.005	0.01	ProductIon
**22:1;O2+2 H_2O**	356.352299	0.005	0.01	ProductIon
**22:0;O2+2 H_2O**	358.367949	0.005	0.01	ProductIon
**2 x Hex**	360.126775	0.005	0.01	NeutralLoss
**2 x Hex**	361.134052	0.005	0.01	ProductIon
**Hex + HexNac**	366.139471	0.005	0.01	ProductIon
**22:1;O3+2 H_2O**	372.347214	0.005	0.01	ProductIon
**22:0;O3+2 H_2O**	374.362864	0.005	0.01	ProductIon
**Hex + HexNac**	383.142759	0.005	0.01	NeutralLoss
**Hex + HexNac**	401.153324	0.005	0.01	NeutralLoss
**Hex + HexNac**	402.160601	0.005	0.01	ProductIon
**2 x HexNac**	407.16602	0.005	0.01	ProductIon
**2 x HexNac**	424.169308	0.005	0.01	NeutralLoss
**2 x HexNac**	442.179873	0.005	0.01	NeutralLoss
**2 x HexNac**	443.18715	0.005	0.01	ProductIon

Table S9: MS/MS search parameters in MS-DIAL for the negative-ion mode.

Species	Mass / u	Tolerance / Da	abundance	Type
**HexNac**	220.08266	0.005	0.001	ProductIon
**Hex**	179.056111	0.005	0.001	ProductIon
**Fucose**	163.061197	0.005	0.001	ProductIon
**NeuAc**	308.098704	0.005	0.001	ProductIon
**NeuGc**	324.093619	0.005	0.001	ProductIon
**2 x HexNac**	441.172597	0.005	0.001	ProductIon
**2 x Hex**	359.119499	0.005	0.001	ProductIon
**Hex + HexNac**	400.146048	0.005	0.001	ProductIon
**HexNac + SO_3 - H**	300.039474	0.005	0.001	ProductIon
**Hex + SO_3 - H**	259.012925	0.005	0.001	ProductIon
**HexNac**	221.089937	0.005	0.001	NeutralLoss
**Hex**	180.063388	0.005	0.001	NeutralLoss
**Fucose**	164.068473	0.005	0.001	NeutralLoss
**NeuAc**	309.10598	0.005	0.001	NeutralLoss
**NeuGc**	325.100895	0.005	0.001	NeutralLoss
**2 x HexNac**	442.179873	0.005	0.001	NeutralLoss
**2 x Hex**	360.126775	0.005	0.001	NeutralLoss
**Hex + HexNac**	401.153324	0.005	0.001	NeutralLoss
**HexNac**	203.079372	0.005	0.001	NeutralLoss
**Hex**	162.052823	0.005	0.001	NeutralLoss

**Fucose**	146.057908	0.005	0.001	NeutralLoss
**NeuAc**	291.095415	0.005	0.001	NeutralLoss
**NeuGc**	307.09033	0.005	0.001	NeutralLoss
**2 x HexNac**	424.169308	0.005	0.001	NeutralLoss
**2 x Hex**	342.11621	0.005	0.001	NeutralLoss
**Hex + HexNac**	383.142759	0.005	0.001	NeutralLoss
**HexNac + SO_3 - H_2O**	283.036185	0.005	0.001	ProductIon
**Hex + SO_3 – H_2O**	242.009636	0.005	0.001	ProductIon
**HexNac**	202.072095	0.005	0.001	ProductIon
**Hex**	161.045546	0.005	0.001	ProductIon
**Fuc**	145.050632	0.005	0.001	ProductIon
**NeuAc**	290.088139	0.005	0.001	ProductIon
**NeuGc**	306.083054	0.005	0.001	ProductIon
**2 x HexNac**	423.162032	0.005	0.001	ProductIon
**2 x Hex**	341.108934	0.005	0.001	ProductIon
**HexNac + Hex**	382.135483	0.005	0.001	ProductIon

Table S10: MS-DIAL settings for the post-identification data analysis. Parameters were optimized to avoid multiple annotations for the same feature while still annotating most of the glycosphingolipids from the database. As both criteria are contrary for the data analysis, the resulting parameters were chosen as a reasonable compromise.

Task	Setting	Value
**Data collection**	MS 1 tolerance	0.05 Da
	MS2 tolerance	0.025 Da
	Retention time begin	0 min
	Retention time range end	50 min
	MS1 mass range begin	500 Da
	MS1 mass rage end	2000 Da
	MS/MS mass range begin	0 Da
	MS/MS mass range end	3000 Da
**Peak detection**	Minimum peak height	10000 amplitude
	Mass slice width	0.25
	Smoothing method	Linear weighted moving average
	Smoothing level	15 scans
	Minimum peak width	5 scans
**Spectrum deconvolution**	Sigma window value	0.5
	MS/MS abundance cut off	0 amplitude
	Exclude after precursor ion	Check
	Keep the isotopic ions until	1
**Identification (GSL database_Post Identification)**	Accurate mass tolerance (MS1)	0.05
	Retention time tolerance	2

	Use retention time for scoring	Check
	Use retention time for filtering	Check
**Alignment parameters**	Reference file	QC_Pos_5
	Retention time tolerance	5 min
	MS1 tolerance	0.05 Da
	Retention time factor	0.5
	MS1 factor	0.5
	Peak count filter	0%
	N% detected in at least one group	80%
	Remove features based on blank information	Check
	Sample max / blank average	5

Table S11: GSL species identified during this study by nano-HILIC MS/MS experiments. An “x” indicates the identification in the corresponding biological group, according to the supplementary note S5.

F. hepatica	**rat liver, acute infection**	**rat liver, control acute infection**	**rat liver, chronic infection**	**rat liver, control chronic infection**	**rat bile duct, chronic infection**	**Glycosphingolipid**
x	x					Hex-Cer 18:0;O2/16:0
x	x					Hex-Cer 18:0;O2/18:0
x	x	x	x			Hex-Cer 18:1;O2/16:0
	x		x	x		Hex-Cer 18:1;O2/24:0
	x				x	Hex-Cer 18:1;O2/24:1
					x	Hex-Cer 20:1;O2/24:1
					x	Hex-Cer 20:1;O2/h24:0
x						Hex-Cer 18:0;O3/16:0
x						Hex-Cer 18:0;O3/18:0
x	x		x	x	x	Hex-Cer 18:0;O3/24:2
x						Hex-Cer 18:0;O3/30:0
x						Hex-Cer18:0;O3/30:1
x						Hex-Cer 18:0;O3/30:2
x				x	x	Hex-Cer 18:0;O3/h16:0
x						Hex-Cer 18:0;O3/h17:0
x			x		x	Hex-Cer 18:0;O3/h18:0
x					x	Hex-Cer 18:0;O3/h24:0
x						Hex-Cer 18:0;O3/h30:0
x						Hex-Cer 18:0;O3/h30:1
x						Hex-Cer 18:0;O3/h30:2
x						Hex-Cer 18:0;O3/h32:1
x						Hex-Cer 18:0;O3/h32:2
x						Hex-Cer 20:0;O3/18:0
x						Hex-Cer 20:0;O3/h18:0

x					x	Hex-Cer 20:0;O3/h24:0
x					x	Hex-Cer 20:0;O3/h24:1
					x	Hex-Cer 20:1;O3/24:1
x	x	x				Hex_2-Cer 18:0;O2/16:0
x	x					Hex_2-Cer 18:0;O2/22:1
x	x	x		x	x	Hex_2-Cer 18:1;O2/16:0
x	x	x	x	x	x	Hex_2-Cer 18:1;O2/24:1
x						Hex_2-Cer 18:0;O3/16:0
			x			Hex_2-Cer 18:0;O3/18:0
x						Hex_2-Cer 18:0;O3/22:2
						Hex_2-Cer 18:0;O3/24:1
x						Hex_3-Cer 37:0;O4
x					x	Hex_3-Cer 42:2;O3
						Hex_3-Cer 44:2;O2
x						Hex_3-Cer 48:1;O3
x						Hex_3-Cer 18:0;O2/16:0
x						Hex_3-Cer 18:0;O2/18:0
x						Hex_3-Cer 18:0;O2/22:2
x					x	Hex_3-Cer 18:0;O2/24:2
x					x	Hex_3-Cer 18:1;O2/24:0
					x	Hex_3-Cer 18:1;O2/24:1
x	x				x	Hex3-Cer 18:1;O2/24:2
x						Hex_3-Cer 18:0;O3/16:0
x				x		Hex_3-Cer 18:0;O3/18:0
x						Hex_3-Cer 18:0;O3/24:2
x						Hex_3-Cer 18:0;O3/h18:0
x						Hex_3-Cer 18:0;O3/h30:0
x						Hex_3-Cer 18:0;O3/h30:2
x						Hex_3-Cer 18:0;O3/h32:2
x				x		Hex_3-Cer 20:0;O3/18:0
x						Hex_3-Cer 20:0;O3/h18:0
						Hex_4-Cer 35:0;O4
x						Hex_5-Cer 42:2;O3
x						Hex_5-Cer 18:0;O2/16:0
x						Hex_5-Cer 18:0;O2/22:2
x						Hex_5-Cer 18:0;O2/26:2
x						Hex_5-Cer 18:1;O2/24:1
x						Hex_5-Cer 18:1;O2/h22:1
x						Hex_6-Cer 40:2;O2
x						Hex_6-Cer 18:0;O2/24:2
x						Hex_6-Cer 18:1;O2/h24:1
x						Hex_6-Cer 18:0;O3/h24:2
						Hex_8-Cer 42:2;O2
					x	$HexNacHex_3$-Cer 34:1;O3
					x	$HexNacHex_3$-Cer 35:1;O2

	x			x	x	HexNacHex$_3$-Cer 41:1;O2
			x		x	HexNacHex$_3$-Cer 42:0;O3
	x	x	x	x	x	HexNacHex$_3$-Cer 18:1;O2/16:0
			x	x	x	HexNacHex$_3$-Cer 18:1;O2/18:0
					x	HexNacHex$_3$-Cer 18:1;O2/20:0
x	x					HexNacHex$_3$-Cer 18:1;O2/22:0
	x	x	x		x	HexNacHex$_3$-Cer 18:1;O2/24:0
	x	x	x	x	x	HexNacHex$_3$-Cer 18:1;O2/24:1
	x			x	x	HexNacHex$_3$-Cer 18:1;O2/24:2
			x			HexNacHex$_3$-Cer 18:1;O2/h24:0
	x	x	x	x	x	HexNacHex$_5$-Cer 18:1;O2/24:1
x					x	HexNac$_2$Hex$_3$-Cer 42:1;O2
						HexNac$_2$Hex$_4$-Cer 18:1;O2/h24:0
x						HexNac$_3$Hex$_3$-Cer 18:0;O2/24:2
x						HexNac$_3$Hex$_3$-Cer 18:1;O2/22:1
x				x		HexNac$_3$Hex$_3$-Cer 18:0;O3/22:2
x			x			HexNac$_3$Hex$_3$-Cer 18:0;O3/24:2
						HexNac$_3$Hex$_5$-Cer 42:3;O2
x						HexNac$_4$Hex$_3$-Cer 18:1;O2/22:1
x						HexNac$_4$Hex$_3$-Cer 18:1;O2/24:1
x						HexNac$_4$Hex$_4$-Cer 18:1;O2/h24:1
x						HexNac$_4$Hex$_4$-Cer 18:0;O3/22:2
x						HexNac$_4$Hex$_4$-Cer 18:0;O3/24:1
x						HexNac$_5$Hex$_3$-Cer 40:1;O2
x						HexNac$_5$Hex$_5$-Cer 18:1;O2/24:1
						HexNac$_5$Hex-Cer 32:0;O2
x						HexNac$_6$Hex$_4$-Cer 40:2;O2
x						HexNac$_6$Hex$_4$-Cer 42:2;O2
x						HexNac$_7$Hex$_4$-Cer 40:2;O2
x						HexNac$_7$Hex$_4$-Cer 42:2;O2
	x				x	HexNacFucHex$_2$-Cer 18:0;O3/h24:0
	x				x	HexNacFucHex$_2$-Cer 18:0;O3/h24:1

	x				x	HexNacFucHex$_3$-Cer 18:1;O2/24:2
					x	HexNacFucHex$_4$-Cer 42:2;O3
	x		x		x	HexNacFucHex$_4$-Cer 18:1;O2/16:0
	x				x	HexNacFucHex$_4$-Cer 18:1;O2/24:0
	x				x	HexNacFucHex$_4$-Cer 18:1;O2/24:1
	x	x	x	x	x	HexNacFucHex$_4$-Cer 18:1;O2/24:2
	x		x	x	x	HexNacFucHex$_4$-Cer 18:1;O2/h24:0
						HexNac$_2$Fuc$_2$Hex$_3$-Cer 18:1;O2/16:0
						HexNac$_2$FucHex$_4$-Cer 18:1;O2/24:2
	x	x	x	x	x	NeuAcHex$_2$-Cer 37:1;O2
	x	x	x	x	x	NeuAcHex$_2$-Cer 39:1;O2
	x	x	x	x	x	NeuAcHex$_2$-Cer 40:2;O2
						NeuAcHex$_2$-Cer 40:3;O2
						NeuAcHex$_2$-Cer 41:2;O2
	x					NeuAcHex$_2$-Cer 42:1;O3
						NeuAcHex$_2$-Cer 42:2;O3
	x	x	x	x	x	NeuAcHex$_2$-Cer 44:2;O2
	x	x	x	x	x	NeuAcHex$_2$-Cer 18:1;O2/16:0
	x	x				NeuAcHex$_2$-Cer 18:1;O2/17:0
	x	x	x	x	x	NeuAcHex$_2$-Cer 18:1;O2/18:0
	x	x	x	x	x	NeuAcHex$_2$-Cer 18:1;O2/20:0
	x	x	x	x	x	NeuAcHex$_2$-Cer 18:1;O2/22:0
	x	x	x	x	x	NeuAcHex$_2$-Cer 18:1;O2/23:0
x	x	x	x	x	x	NeuAcHex$_2$-Cer 18:1;O2/24:0
	x	x	x	x	x	NeuAcHex$_2$-Cer 18:1;O2/24:1
	x	x	x	x	x	NeuAcHex$_2$-Cer 18:1;O2/24:2
	x		x		x	NeuAcHex$_2$-Cer 18:1;O2/h16:0
		x	x	x	x	NeuAcHex$_2$-Cer 18:0;O3/h16:0
					x	NeuAcHex$_2$-Cer 18:0;O3/h24:0
					x	NeuAcHex$_2$-Cer 20:0;O3/h24:0
		x	x	x		NeuGcHex$_2$-Cer 18:1;O2/16:0

		x	x	x	x	NeuGcHex$_2$-Cer 18:1;O2/24:0
					x	NeuGcHex$_2$-Cer 18:1;O2/24:1
	x		x		x	HexNacNeuAcHex$_4$-Cer 34:1;O2
						HexNacNeuAcHex$_4$-Cer 36:1;O2
					x	HexNacNeuAcHex$_4$-Cer 38:1;O2
					x	HexNacNeuAcHex$_4$-Cer 41:1;O2
					x	HexNacNeuAcHex$_4$-Cer 18:1;O2/24:0
					x	HexNacNeuAcHex$_4$-Cer 18:0;O3/h22:0
		x	x	x		HexNacNeuGc$_2$Hex$_3$-Cer 36:1;O2
	x	x	x	x	x	HexNacNeuGc$_2$Hex$_3$-Cer 42:2;O2
	x				x	HexNacNeuGcHex$_3$-Cer 42:3;O2
					x	HexNacNeuGcHex$_4$-Cer 34:1;O2
					x	HexNacNeuGcHex$_4$-Cer 42:1;O2
					x	HexNacNeuGcHex$_4$-Cer 42:1;O3
					x	HexNacNeuGcHex$_4$-Cer 42:2;O2
					x	HexNacNeuGcHex$_4$-Cer 44:1;O4
	x	x				HexNacNeuAc$_2$Hex$_3$-Cer 34:1;O3
	x	x		x	x	HexNacNeuAc$_2$Hex$_3$-Cer 36:1;O2
	x	x	x	x		HexNacNeuAc$_2$Hex$_3$-Cer 38:1;O2
						HexNacNeuAc$_2$Hex$_3$-Cer 38:1;O2
	x	x	x			HexNacNeuAc$_2$Hex$_3$-Cer 40:3;O2
						HexNacNeuAc$_2$Hex$_3$-Cer 42:1;O4
	x	x	x		x	HexNacNeuAc$_2$Hex$_3$-Cer 18:1;O2/16:0)
	x	x	x	x		HexNacNeuAc$_2$Hex$_3$-Cer 18:1;O2/22:0
	x	x	x	x	x	HexNacNeuAc$_2$Hex$_3$-Cer 18:1;O2/22:1
			x			HexNacNeuAc$_2$Hex$_3$-Cer 18:1;O2/23:0
	x		x	x	x	HexNacNeuAc$_2$Hex$_3$-Cer 18:1;O2/24:0
x	x	x	x	x		HexNacNeuAc$_2$Hex$_3$-Cer 18:1;O2/24:1

	x	x	x	x	x	$HexNacNeuAc_2Hex_3$-Cer 18:1;O2/24:2
						$HexNacNeuAc_2Hex_3$-Cer 18:1;O2/h22:1
x					x	$HexNacNeuAc_2Hex_3$-Cer 18:1;O2/h24:1
	x			x	x	$HexNacNeuAc_2Hex_4$-Cer 18:1;O2/16:0
	x	x	x	x	x	$HexNacNeuAc_2Hex_4$-Cer 18:1;O2/24:1
	x		x	x	x	$HexNacNeuAc_3Hex_3$-Cer 36:1;O2
	x	x	x	x	x	$HexNacNeuAc_3Hex_3$-Cer 38:1;O2
	x	x	x	x	x	$HexNacNeuAc_3Hex_3$-Cer 18:1;O2/16:0
	x	x	x	x	x	$HexNacNeuAc_3Hex_3$-Cer 18:1;O2/22:0
	x	x	x	x	x	$HexNacNeuAc_3Hex_3$-Cer 18:1;O2/22:1
	x	x	x	x	x	$HexNacNeuAc_3Hex_3$-Cer 18:1;O2/23:0
	x	x	x	x	x	$HexNacNeuAc_3Hex_3$-Cer 18:1;O2/24:0
	x	x	x	x	x	$HexNacNeuAc_3Hex_3$-Cer 18:1;O2/24:1
					x	$HexNacNeuAc_3Hex_3$-Cer 18:1;O2/h24:1
	x	x	x	x	x	$HexNacNeuAc_4Hex_3$-Cer 18:1;O2/23:0
	x	x	x	x	x	$HexNacNeuAc_4Hex_3$-Cer 18:1;O2/24:0
	x	x	x	x	x	$HexNacNeuAc_4Hex_3$-Cer 18:1;O2/24:1
x						Hex-HPO_3-Cer 18:1;O2/22:2
x						Hex-HPO_3-Cer 18:1;O2/24:2
x						Hex-HPO_3-Cer 19:1;O2/18:0
x						Hex-HPO_3-Cer 20:1;O2/18:0
x						Hex-HPO_3-Cer 18:0;O3/16:0
x						Hex-HPO_3-Cer 18:0;O3/16:1
x						Hex-HPO_3-Cer 18:0;O3/17:0
x	x	x	x		x	Hex-HPO_3-Cer 18:0;O3/18:0
						Hex-HPO_3-Cer 18:0;O3/18:1
x						Hex-HPO_3-Cer 18:0;O3/28:1
x				x		Hex-HPO_3-Cer 18:0;O3/30:2
x						Hex-HPO_3-Cer 18:0;O3/h28:0
x					x	Hex-HPO_3-Cer 18:0;O3/h30:0
x						Hex-HPO_3-Cer 18:0;O3/h30:1
x						Hex-HPO_3-Cer 18:0;O3/h32:2

x						Hex-HPO_3-Cer 19:0;O3/h18:0
x				x		Hex-HPO_3-Cer 20:0;O3/18:0
x						Hex-HPO_3-Cer 20:0;O3/h18:0
x						Hex-HPO_3-Hex-Cer 34:0;O4
x				x		Hex-HPO_3-Hex-Cer 18:0;O3/18:0
x	x		x	x	x	Hex-HPO_3-Hex-Cer 18:0;O3/h18:0
					x	HexNac-HPO_3-Hex-Cer 42:0;O4
x						HexNac-HPO_3-Hex-Cer 18:1;O2/18:0
						HexNac-HPO_3-Hex-Cer 18:0;O3/16:0
x						HexNac-HPO_3-Hex-Cer 18:0;O3/17:0
x	x					HexNac-HPO_3-Hex-Cer 18:0;O3/18:0
x						HexNac-HPO_3-Hex-Cer 18:0;O3/28:1
x	x					HexNac-HPO_3-Hex-Cer 18:0;O3/30:2
x						HexNac-HPO_3-Hex-Cer 18:0;O3/h16:0
x				x		HexNac-HPO_3-Hex-Cer 18:0;O3/h18:0
x						HexNac-HPO_3-Hex-Cer 18:0;O3/h30:2
x						HexNac-HPO_3-Hex-Cer 18:0;O3/h32:2
x						HexNac-HPO_3-Hex-Cer 19:0;O3/h18:0
x						HexNac-HPO_3-Hex-Cer 20:0;O3/18:0
x				x		HexNac-HPO_3-Hex-Cer 20:0;O3/h18:0

Table S12: Significant up- or downregulated species in the chronic infection stage compared to the same-aged healthy control. The RP-LC MS/MS data was analyzed according to the Supplementary Note 3. For revealing significant up- and downregulation between two biological groups, a two-sided Student’s T-tests with a threshold p-value of 0.05 was performed. “↑” indicates upregulated species in the chronic infection, and “↓” downregulated species, respectively.

**Metabolite Name**	**Up- or downregulated at the chronic infection stage**	**p-value**
CAR 16:1	↓	0.01554072
DG 32:4	↑	0.00125331
DG 52:4|DG 16:0_36:4	↓	0.02533482
LPC O-16:0	↑	0.03285521
LPC O-16:1	↑	0.01026609
LPE O-18:1	↑	0.03906403
PEtOH 34:2|PEtOH 16:0_18:2	↑	0.03262315
RIKEN P-VS1 ID-9947 from Mouse_Eye_fads2KO_N_Ctr	↓	0.01181219
ST 24:1;O5	↑	0.00626699
TG 62:14|TG 18:2_22:6_22:6	↓	0.03770863
TG 62:14|TG 20:4_20:4_22:6	↓	0.04641163

References

(1) Paschke, C.; Leisner, A.; Hester, A.; Maass, K.; Guenther, S.; Bouschen, W.; Spengler, B. Mirion--a software package for automatic processing of mass spectrometric images, *Journal of the American Society for Mass Spectrometry.* **2013**, *24,* pp. 1296–1306.

(2) Goedhart, J.; Luijsterburg, M. S. VolcaNoseR is a web app for creating, exploring, labeling and sharing volcano plots, *Scientific reports.* **2020**, *10,* p. 20560.

(3) Metsalu, T.; Vilo, J. ClustVis: a web tool for visualizing clustering of multivariate data using Principal Component Analysis and heatmap, *Nucleic acids research.* **2015**, *43,* W566-70.
